# Analyses of the three 1-Cys Peroxiredoxins from *Aspergillus fumigatus* reveal that cytosolic Prx1 is central to H_2_O_2_ metabolism and virulence

**DOI:** 10.1038/s41598-018-30108-2

**Published:** 2018-08-17

**Authors:** Marina Campos Rocha, Krissia Franco de Godoy, Renata Bannitz-Fernandes, João H. T. Marilhano Fabri, Mayra M. Ferrari Barbosa, Patrícia Alves de Castro, Fausto Almeida, Gustavo Henrique Goldman, Anderson Ferreira da Cunha, Luis E. S. Netto, Marcos Antonio de Oliveira, Iran Malavazi

**Affiliations:** 1 Departamento de Genética e Evolução, Centro de Ciências Biológicas e da Saúde, Universidade Federal de São Carlos, São Carlos, SP 13.565-905 Brazil; 2 Departamento de Genética e Biologia Evolutiva, Instituto de Biociências, Universidade de São Paulo, São Paulo, SP 05508-090 Brazil; 3 Departamento de Ciências Farmacêuticas, Faculdade de Ciências Farmacêuticas de Ribeirão Preto, Universidade de São Paulo, Ribeirão Preto, SP 14.040-903 Brazil; 4 Departamento de Bioquímica e Imunologia, Faculdade de Medicina de Ribeirão Preto, Universidade de São Paulo, Ribeirão Preto, SP 14.040-900 Brazil; 5 Instituto de Biociências, Universidade Estadual Paulista, Campus do Litoral Paulista, São Vicente, SP 11.380-972 Brazil; 6 Present Address: Instituto Butantan, São Paulo, SP 05503-900 Brazil

## Abstract

Standing among the front defense strategies against pathogens, host phagocytic cells release various oxidants. Therefore, pathogens have to cope with stressful conditions at the site of infection. Peroxiredoxins (Prx) are highly reactive and abundant peroxidases that can support virulence and persistence of pathogens in distinct hosts. Here, we revealed that the opportunistic human pathogen *A*. *fumigatus* presents three 1-Cys Prx (Prx6 subfamily), which is unprecedented. We showed that PrxB and PrxC were in mitochondria, while Prx1 was in cytosol. As observed for other Prxs, recombinant Prx1 and PrxC decomposed H_2_O_2_ at elevated velocities (rate constants in the 10^7^ M^−1^s^−1^ range). Deletion mutants for each Prx displayed higher sensitivity to oxidative challenge in comparison with the wild-type strain. Additionally, cytosolic Prx1 was important for *A*. *fumigatus* survival upon electron transport dysfunction. Expression of Prxs was dependent on the SakA^HOG1^ MAP kinase and the Yap1^YAP1^ transcription factor, a global regulator of the oxidative stress response in fungi. Finally, cytosolic Prx1 played a major role in pathogenicity, since it is required for full virulence, using a neutropenic mouse infection model. Our data indicate that the three 1-Cys Prxs act together to maintain the redox balance of *A*. *fumigatus*.

## Introduction

*Aspergillus fumigatus* is a mold with a notorious ability to infect immunocompromised individuals and therefore cause severe systemic infections in this cohort of patients. *A*. *fumigatus* solely accounts for approximately 90% incidence of the invasive pulmonary aspergillosis (IPA) cases worldwide. Mortality rates associated with IPA can be as high as 50% even if properly diagnosed and treated. However, if the diagnosis is not reached or is delayed, death can occur in nearly 100% of infected individuals^1,2^.

Among the front defense strategies against pathogens, host effector immune cells release oxidants, such as reactive oxygen species (ROS) and reactive nitrogen species (RNS). This process ultimately generates oxidative/nitrosative stresses to the pathogen at the infection site^3^. At high levels, ROS and RNS cause damage to the macromolecules of invading microorganisms^4^. Furthermore, pathogens must also metabolize ROS produced by its own metabolism. To detoxify the ROS, which include several hydroperoxide species such as H_2_O_2_ and organic hydroperoxides, numerous complex mechanisms have evolved in pathogens^5–8^. Therefore, hydroperoxide scavenging enzymes are highly relevant for the success of pathogen adaptation and colonization of the host tissues. Moreover, low-level production of hydroperoxides is also implied in fungal cell growth and differentiation^3,9^. In this sense, antioxidant proteins in pathogens may regulate hydroperoxides in suitable levels to allow cell growth and differentiation^10^.

Among such defense mechanisms, peroxiredoxins (Prxs) are Cys-based peroxidases that play pivotal roles to support the virulence and persistence of several microorganisms in distinct hosts^11–16^. Prxs are abundant, highly reactive and specific towards oxidants, such as H_2_O_2_, lipid hydroperoxides and peroxynitrite (*k* = 10^6^–10^8^ M^−1^.s^−1^)^17–19^. Moreover, Prxs can be found in the cytosol, mitochondria, nuclei and peroxisomes and are associated in the membranes or even secreted to the extracellular space^20,21^. Although all Prxs have a fully conserved Cys residue, the so-called peroxidatic cysteine (C_P_), directly involved in the reduction of hydroperoxides, the Prx family presents high functional and structural diversity. While some Prxs have only the C_P_ , others have a second residue capable of forming a disulfide bond with C_P_ , called the resolution cysteine (C_R_). Based on this catalytic mechanism, Prx enzymes are commonly classified as 1-Cys, when only one cysteine is involved in the catalytic cycle, or 2-Cys, when the C_R_ is present^22,23^.

Comparatively, 1-Cys Prxs are less studied than the 2-Cys counterparts. Notably, 1-Cys Prx from mammals (Prdx6) is a dual-function enzyme with both peroxidase and acidic Ca^2+^-independent phospholipase A2 activities^24^. This additional function is thought to protect cell membrane phospholipids against peroxidation and hydrolysis^25^. Remarkably, some 1-Cys Prxs from pathogens have distinguishing features in comparison with the corresponding host enzymes, placing them as promising targets for the development of specific drugs^11,19,26–28^. For instance, a 1-Cys Prx from the plant fungal pathogen *Magnaporthe oryzae* and from the mammalian bacterial pathogen *Pseudomonas aeruginosa* are necessary to decompose ROS generated by the host and therefore important for their virulence^12,13^.

In this study, we describe for the first time an organism that presents three 1-Cys Prx enzymes, all of them belonging to the Prx6 sub-family^29^. In contrast, most organisms present only one gene for 1-Cys Prx in their genomes. To gain insights in this unique repertoire of antioxidant enzymes, we performed several genetic and biochemical analysis of Prx1, PrxB and PrxC enzymes. Among other aspects, we observed that two of the 1-Cys Prx are mitochondrial enzymes (PrxB and PrxC), whereas the other is cytosolic (Prx1). Furthermore, the expression of the three 1-Cys Prxs is regulated by the signaling pathways involving the SakA^HOG1^ MAP kinase and Yap1^YAP1^ transcription factor. Finally, cytosolic Prx1 is the main 1-Cys Prx involved in the tolerance of *A*. *fumigatus* to oxidative damage, playing a major role in pathogenicity.

## Results

### The *A*. *fumigatus* genome has three 1-Cys Prx genes

Initially, the repertoire of antioxidant enzymes in the *A*. *fumigatus* A1163 genome was analyzed using Prx enzymes from *Saccharomyces cerevisiae* and *Homo sapiens* as query sequences. This analysis revealed that *A*. *fumigatus* possesses six putative Prx-encoding genes. Three of them encoding atypical 2-Cys Prxs (having an intramolecular disulfide as a catalytic intermediate): AFUB_049980, AFUB_063010 and the AFUB_096050^14^. In contrast, no typical 2-Cys Prx (Prx1/AhpC subgroup^29^) was found. We also identified three genes encoding 1-Cys isoforms: AFUB_065670, AFUB_062560 and AFUB_080670. Notably, this is the first description of a genome containing three genes encoding 1-Cys Prxs that belong to the Prx6 subgroup^29^. We named AFUB_062560 and AFUB_080670 as *prxB* and *prxC*, respectively to be consistent with *A*. *fumigatus* nomenclature. The gene AFUB_065670 was previously named as *prx1*^30^.

We focused our studies on the three 1-Cys Prx enzymes, since they are less studied than the 2-Cys Prx counterparts. Moreover, the number of these isoforms found in the *A*. *fumigatus* genome is unprecedented, frequently only one 1-Cys Prx isoform or none is found in the majority of organisms. Our results revealed that except for *A*. *fumigatus*, all *Aspergillus* species available in the *Aspergillus* Genome Database (http://www.aspergillusgenome.org) possess at least two isoforms of 1-Cys Prx with amino acid identity raging from 70–90% with the *A*. *fumigatus* 1-Cys Prx counterparts. In each species, one of the two isoforms presented a putative N-terminal mitochondrial target sequence (probability > 90%). Noteworthy, only in *A*. *fumigatus* strains (A1163 and Af293), three 1-Cys Prx isoforms were detected. These data reinforces that the number of isoforms of 1-Cys Prx is unusual in *Aspergilli*, especially in *A*. *fumigatus*.

**Figure 1:**
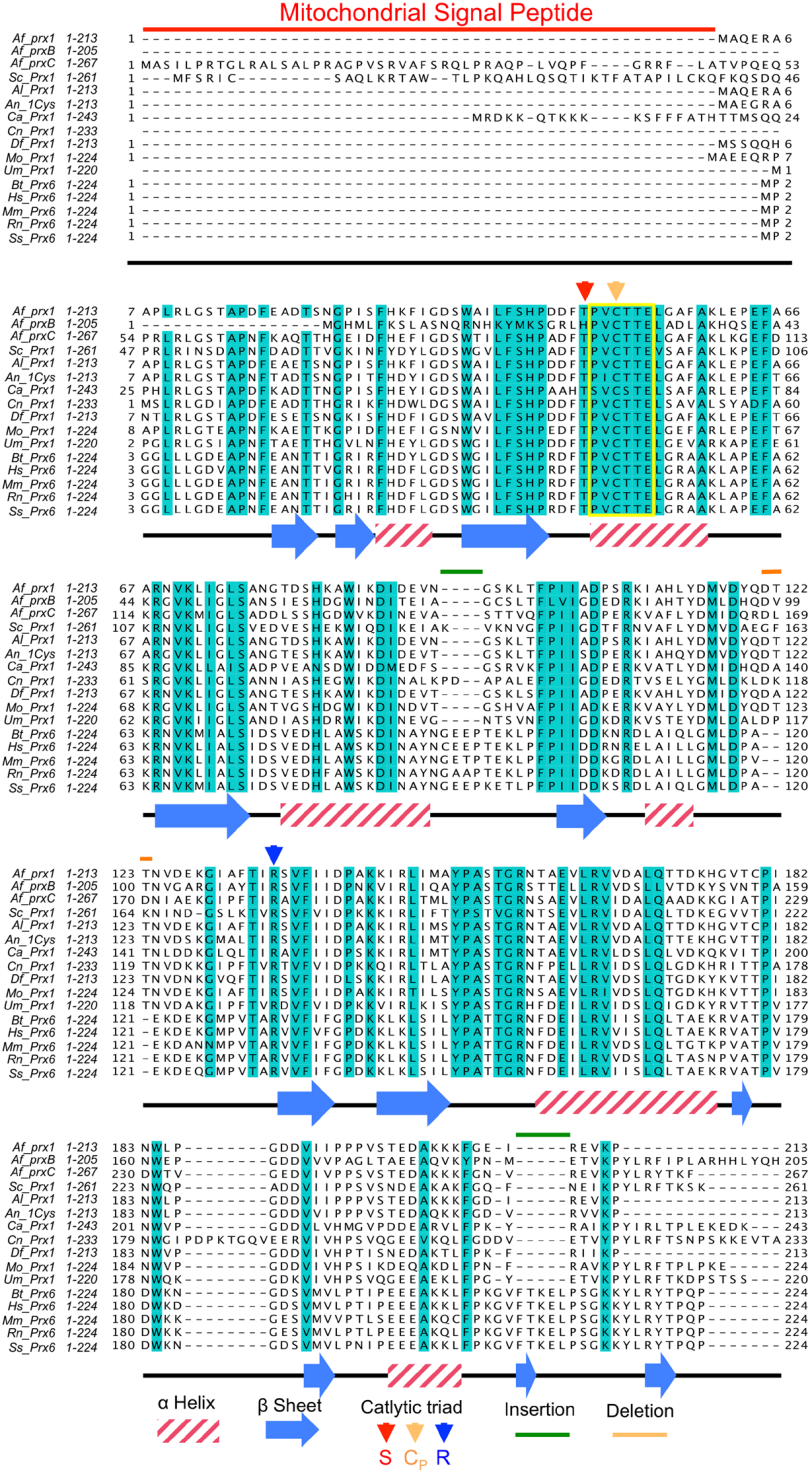
Sequence alignment of 1-Cys Prx from *A*. *fumigatus*, other fungi and mammals. Identical residues among sequences are depicted in blue. The yellow box denotes the conserved motif PVC_P_TTE in the Prx6 sub-class. The colored arrowheads indicate the conserved amino acids of the catalytic triad: Thr (red), C_P_ (orange) and Arg (blue). The colored bars denote the mitochondria signal peptide (red), residues insertions (green) or deletions (orange). The secondary structure assignments based on the structure of *H*. *sapiens* Prx6 (PDB code: 1PRX) are shown below the amino acid sequences. The species abbreviation initials are given in italics and are as follows: *Af_prx1* = *A*. *fumigatus prx1* (AFUB_065670); *Af_prxB* = *A*. *fumigatus prxB* (AFUB_062560); *Af_prxC* = *A*. *fumigatus prxC* (AFUB_080670); *Sc_Prx1* = *S*. *cerevisiae* Prx1 (NP_009489.1); *Al_Prx1* = *A*. *lentulus* Prx1 (GAQ08014.1); *An_Prx1* = *A*. *nidulans* 1-Cys peroxiredoxin (CBF85378.1); *Ca_Prx1* = *Candida albicans* putative Prx1 (XP_716935.1); *Cn_Prx1* = *Cryptococcus neoformans* Prx (XP_567781); *Df_Prx1* = *Debaryomyces fabryi* Prx (XP_015465609.1); *Mo_Prx1* = *Magnaporthe oryzae* mitochondrial peroxiredoxin Prx1 (XP_003715830.1); *Um_Prx1* = *Ustilago maydis* Prx (XP_011392689); *Hs_Prx6* = *Homo sapiens* Prx6 (NP_004896.1); *Bt_Prx6* = *Bos taurus* Prx6 (NP_777068.1); *Mm_Prx6* = *Mus musculus* Prx6 (NP_031479.1); *Rn_Prx6* = *Rattus norvegicus* Prx6 (NP_446028.1); *Ss_Prx6* = *Sus scrofa* Prx6 (NP_999573.1).

The three *A*. *fumigatus* 1-Cys Prx genes presented moderate similarity with the human orthologue (Prx6), ranging from 37% (*prx1*) to 51% identity (*prxB* and *prxC*). In addition, they contain a C_P_ embedded in the highly conserved PVC_P_TTE motif, which is archetypal to the Prx6 subclass (Fig. 1)^25,29,31^.

All the Prx enzymes characterized to date present a catalytic triad that also comprises a Thr (in some cases replaced by a Ser) and an Arg residue^18^. As expected, these three residues are conserved in Prx1 and PrxC, but surprisingly, the catalytic triad Thr is substituted by a His residue in the primary sequence of PrxB. Unfortunately, we were unable to obtain the soluble form of recombinant PrxB to investigate the ability of this enzyme to reduce hydrogen peroxide (see below). PrxC presents a N-terminal extension (Fig. 1), suggesting mitochondrial localization, as is seen for the 1-Cys Prx from *S*. *cerevisiae* (Prx1)^32^. Indeed, PrxC displayed a high probability (0.99) of being targeted to mitochondria with the predicted cleavage site located at the residue 59.

### *A*. *fumigatus* Prx1 and PrxC are able to efficiently decompose H_2_O_2_

**Figure 2:**
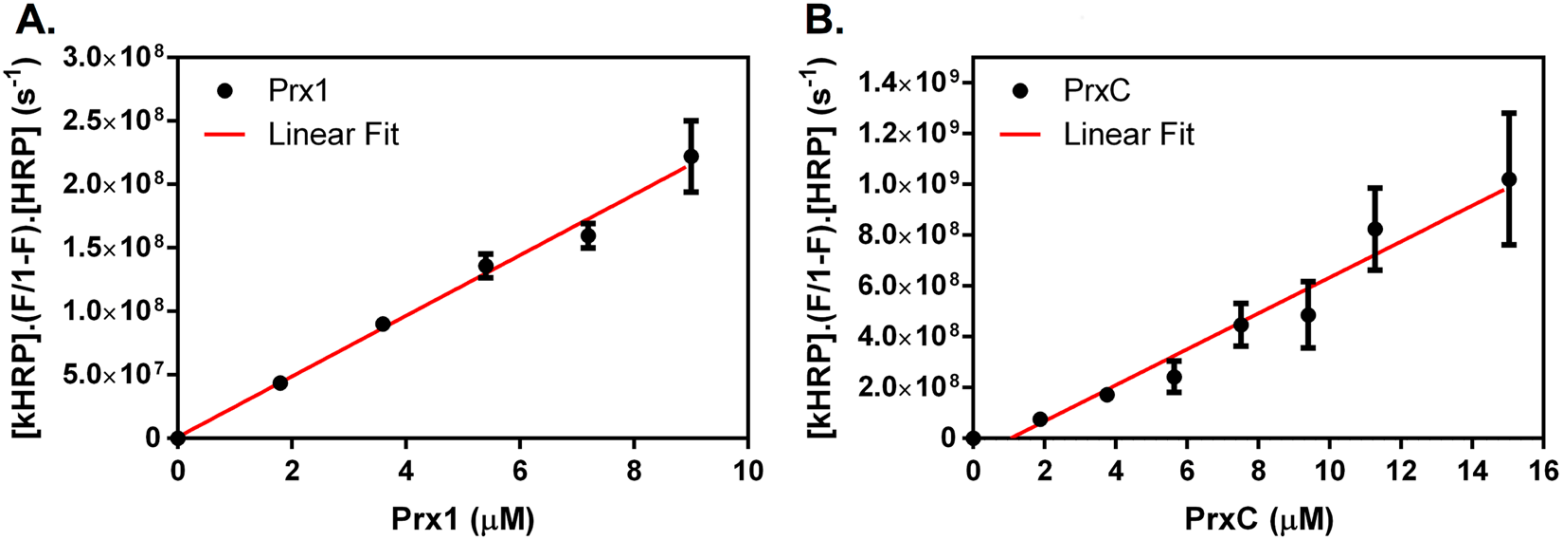
Oxidation rates of *A*. *fumigatus* Prx1 and PrxC. The second-order rate constants for the reactions of Prx1 (**A**) and PrxC (**B**) were determined using growing concentrations of Prx (2–16 μM) with H_2_O_2_ (4 μM) by the competition with HRP (horseradish peroxidase) (8 μM). The rates were determined as *k*_Prx1_ = 2.28 × 10^7^ M^−1^s^−1^ and *k*_PrxC_ = 7.07 × 10^7^ M^−1^s^−1^. The left y-axis is relative to the enzyme activity and the x-axis is relative to the enzyme concentration added to the reaction. The results shown are mean ± SD obtained from three independent replicates.

Next, we attempted to express the three 1-Cys Prx enzymes in *Escherichia coli* strains, but after several trials no soluble PrxB was obtained, precluding further characterization. In contrast, Prx1 and PrxC were successfully expressed and purified (Supplementary Fig. S1). Then, their ability to decompose H_2_O_2_ was analyzed by a competitive assay against horseradish peroxidase (HRP). The rate constants for the reactions of Prx1 and PrxC with H_2_O_2_ were both in the 10^7^ M^−1^s^−1^ range (Fig. 2), therefore indicating that these two enzymes are highly efficient peroxidases. Their abilities to reduce H_2_O_2_ were similar to other Prxs previously described^21,33,34^ and comparable to catalases and glutathione peroxidases^35,36^. Original absorbance spectra for the HRP competitive assay are available in Supplementary Figure S2.

Unfortunately, we were unable to identify reductants for Prx1 and PrxC, which preclude as from performing bi-substrate, steady-state kinetics. Preliminary studies indicated that thioredoxin and GSH are poor reducing substrates for these peroxidases.

### 1-Cys Prxs differentially contribute to oxidative challenge tolerance

Subsequently, *prx1*, *prxB* and *prxC* were deleted (Supplementary Fig. S3A-K) and phenotypes were investigated. Each individual gene deletion strain was also complemented with the corresponding wild-type allele to discard the occurrence of possible secondary mutations. The mRNA levels for each Prx were assessed by RT-qPCR. As expected, they were similar in the complemented and wild-type strains, while absent in the corresponding null mutant (data not shown).

We also attempted to isolate double and triple mutants for *prx1*, *prxB* and *prxC* genes, but we only succeeded in obtaining the Δ*prxB* Δ*prxC* double mutant (Supplementary Fig. S3M). Remarkably, any double mutant containing the deletion of the *prx1* gene seemed to be non-viable when we platted the transformed protoplasts in the regeneration medium (YG supplemented with 0.6 M KCl as osmotic stabilizer). Therefore, we isolated a conditional mutant for *prx1* under the control of the nitrate reductase promoter (*niiA*)^37^ to be used in the construction of double mutants. However, under repressive conditions (Minimal Medium supplemented with 50 mM ammonium tartrate), the *niiA::prx1* mutant did not exhibit phenotypes similar to the null mutant Δ*prx1* in the presence of oxidants (data not shown). Possibly, transcriptional repression achieved by the *niiA* promoter was not strong enough to generate a loss-of-function phenotype. Further experimentation is required to understand the essentiality of the *prx1* gene in backgrounds already deficient for the other 1-Cys Prxs.

**Figure 3:**
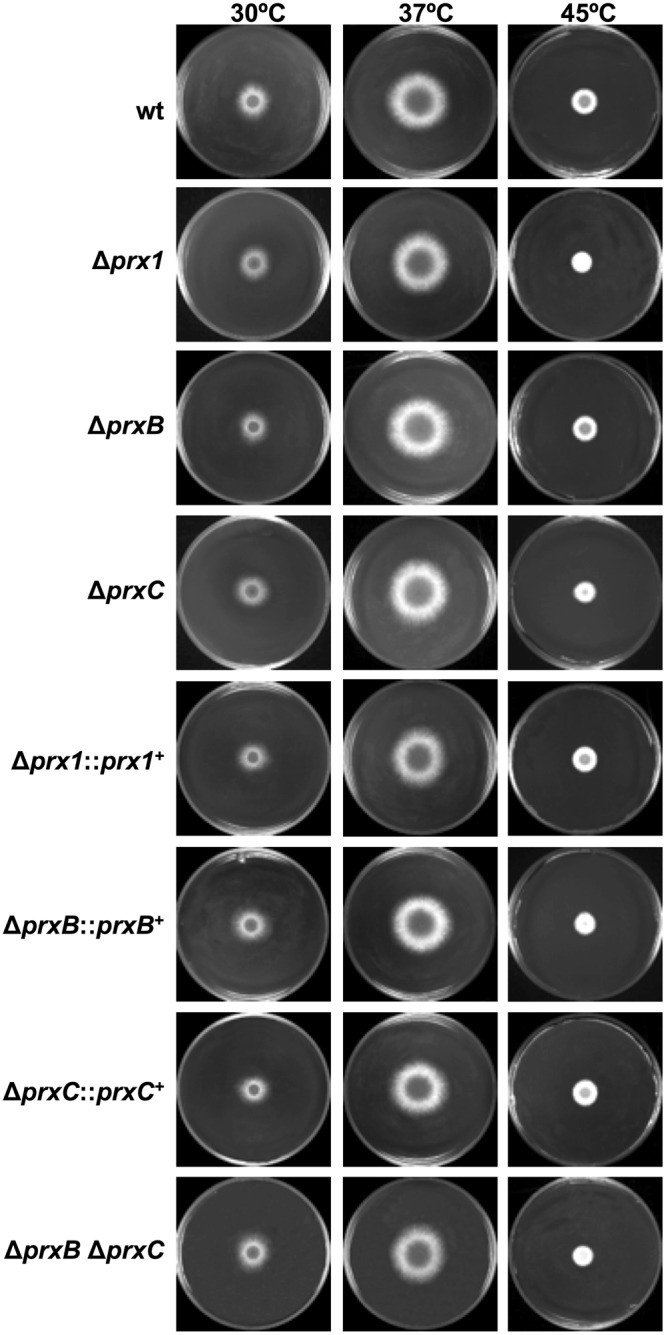
Radial growth of the wild-type and Prx null mutants on MM. 1 × 10^4^ conidia of each strain were inoculated onto the center of solid MM and incubated at the indicated temperatures for 72 h. Results are representative of triplicate experiments with similar results.

**Figure 4:**
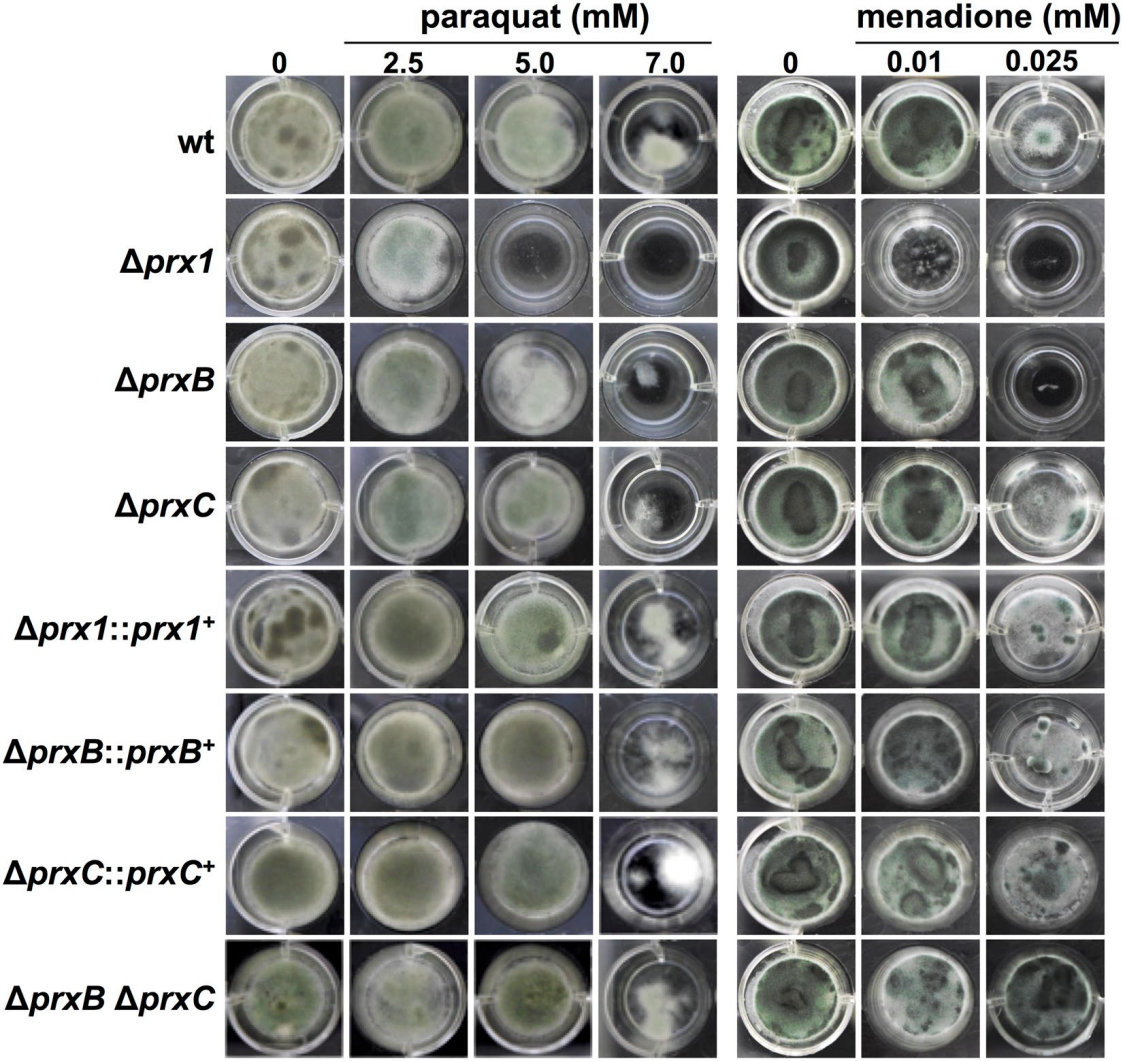
Prx null mutants are sensitive to oxidative-damaging agents. 1 × 10^4^ conidia of wild-type, mutant and complemented strains were inoculated in 1 ml of MM (24 well plates) supplemented or not with varying concentration of paraquat and menadione. Plates were incubated at 37 °C for 72 h and then photographed. Experiments were repeated three times and a representative result is shown.

Next, phenotypes were evaluated in cells grown in minimal medium (MM). In non-stressing conditions, the deletion mutants displayed radial growth comparable to the wild-type and complemented strains (Fig. 3). The growth kinetics, germination rates and conidiation levels were also analyzed but no significant differences were observed for the three 1-Cys Prx null mutants in comparison to the wild-type strain (data not shown). However, the three Prx null mutants displayed increased sensitivity to paraquat and menadione (Fig. 4 and Supplementary Fig. S4), two compounds that continuously generated superoxide and H_2_O_2_ by the redox cycling. Surprisingly, the tolerance of the double mutant Δ*prxB* Δ*prxC* to paraquat was comparable to the wild-type strain, suggesting compensatory effects such as increased expression of other antioxidant enzyme. As a general trend, the sensitivities to oxidative challenge were more evident for the Δ*prx1* mutant.

In contrast, the three Prx mutants were as resistant as wild-type cells to exogenous H_2_O_2_ treatment (Supplementary Fig. S5). Possibly, the three 1-Cys Prx enzymes might be more adapted to the removal of H_2_O_2_ continuously formed by the autoxidation of redox compounds, such as paraquat, menadione (Fig. 4) and components of the respiratory chain.

Since glucose represses the expression of genes involved in mitochondrial biogenesis, respiratory metabolism and antioxidant defense in *S*. *cerevisiae*^38^, we hypothesized that sensitivity to H_2_O_2_ would be altered in cells growing in non-fermentative carbon sources, such as glycerol and lactate. However, again, no increased sensitivity was observed, even in the double mutant Δ*prxB* Δ*prxC* (Supplementary Fig. S5A-B).

Additionally, *prx1*, *prxB* and *prxC* were dispensable for voriconazole and caspofungin susceptibility (data not shown) while a slight sensitivity to amphotericin B and SDS was observed for the Δ*prx1* mutant (Supplementary Fig. S6).

In summary, our phenotypic analysis indicated that all three 1-Cys Prx are relevant in conditions where H_2_O_2_ is continuously formed by the redox cycling of paraquat and menadione and Δ*prx1* is more sensitive to these stressful conditions than the other two deletion mutants.

### Sub-cellular localization of *A*. *fumigatus* 1-Cys Prxs

**Figure 5:**
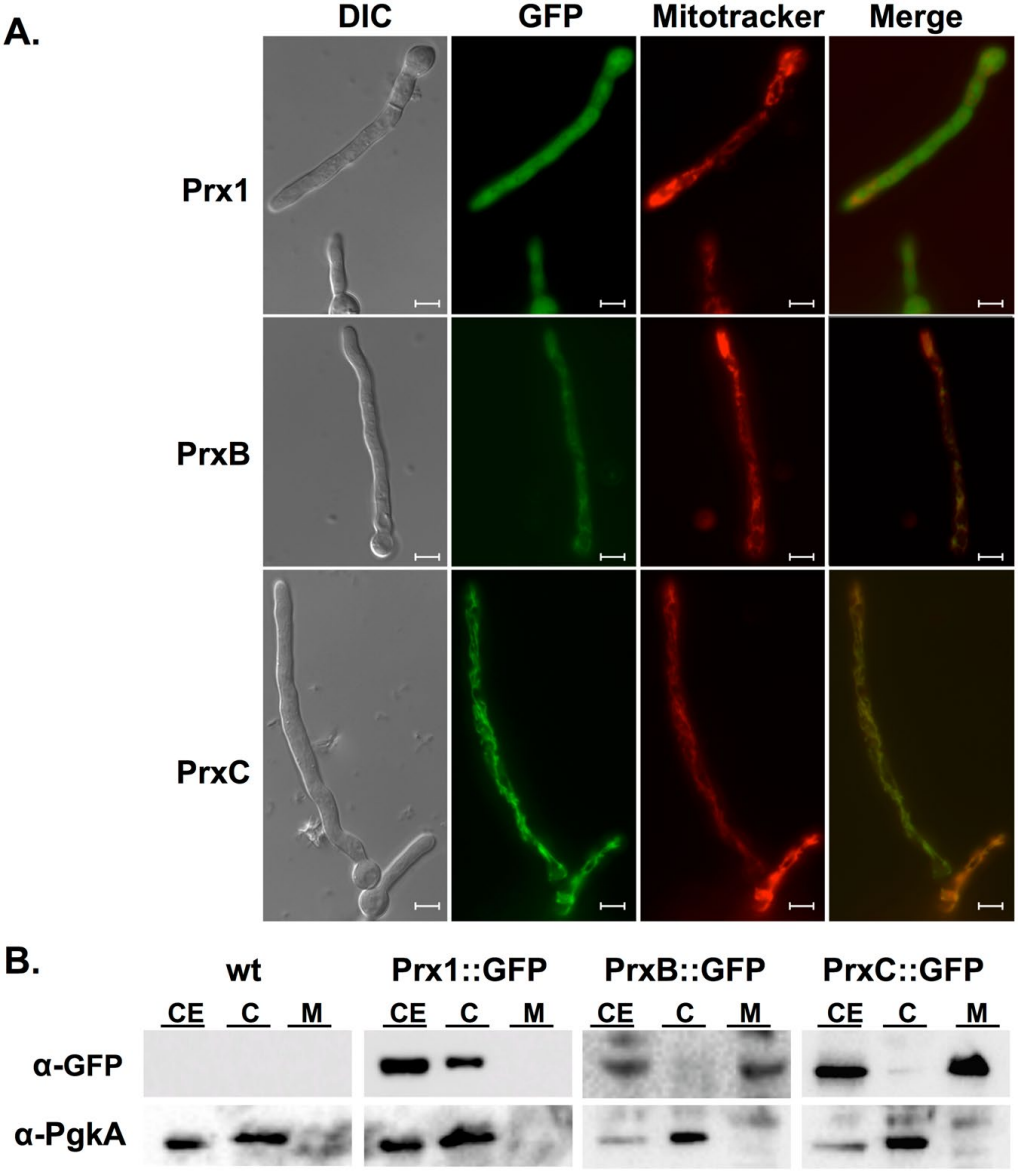
Prx1 localizes to the cytosol, while PrxB and PrxC are mitochondrial Prxs. (**A**) The Prx::GFP strains were grown for 16 h at 30 °C in MM, stained with Mitotracker, washed and directly inspected under the fluorescence microscope. Bars: 5 µm. (**B**) Western blot of the cytosolic and mitochondrial fractions of the Prx::GFP strains. CE: crude extract; C: cytosolic fraction; M: mitochondrial fraction. Prxs were detected by using an anti-GFP antibody. An anti-PgkA antibody served as fractionation control. Experiments were repeated three times and a representative result is shown. In the figure are reported the cropped gels/blots obtained by each protein evaluation. All gels were run in the same experimental conditions. Full-length blots are presented in Supplementary Figure S8.

To assess the subcellular localization of *A*. *fumigatus* Prxs, we constructed the strains expressing Prx1, PrxB and PrxC fused to GFP and under the control of their native promoters (Supplementary Fig. S3N-S). The sensitivities of these strains to oxidative insult were comparable to the wild-type strain indicating that they are fully functional (Supplementary Fig. S7). The Prx1::GFP strain produced a strong and diffuse fluorescent signal without a noticeably sub-cellular accumulation, consistent with a cytosolic localization (Fig. 5). In contrast, the fluorescence of PrxB-GFP co-localized with the mitochondrial network, but the signal intensity was weaker than that of Prx1-GFP and PrxC-GFP. The PrxC-GFP fusion protein produced the most intense fluorescent signal, which also co-localized with Mitotracker, being evenly distributed along mitochondria (Fig. 5A). This was expected, since as mentioned above, the product of the *prxC* gene presents a predicted presequence signal for mitochondrial localization (Fig. 1). As an alternative method, we performed cell fractionation of the mycelium and samples were then analyzed by Western blot using anti-GFP to detect each of the 1-Cys Prx. According to the microscopic studies, Prx1 was detected in the cytosolic fractions, while PrxB and PrxC were present in the mitochondrial fractions (Fig. 5B). Therefore, the three 1-Cys Prxs are abundant in the cytosol and mitochondrial compartments even in the absence of exogenous oxidative challenge.

### Expression patterns of 1-Cys Prxs

**Figure 6:**
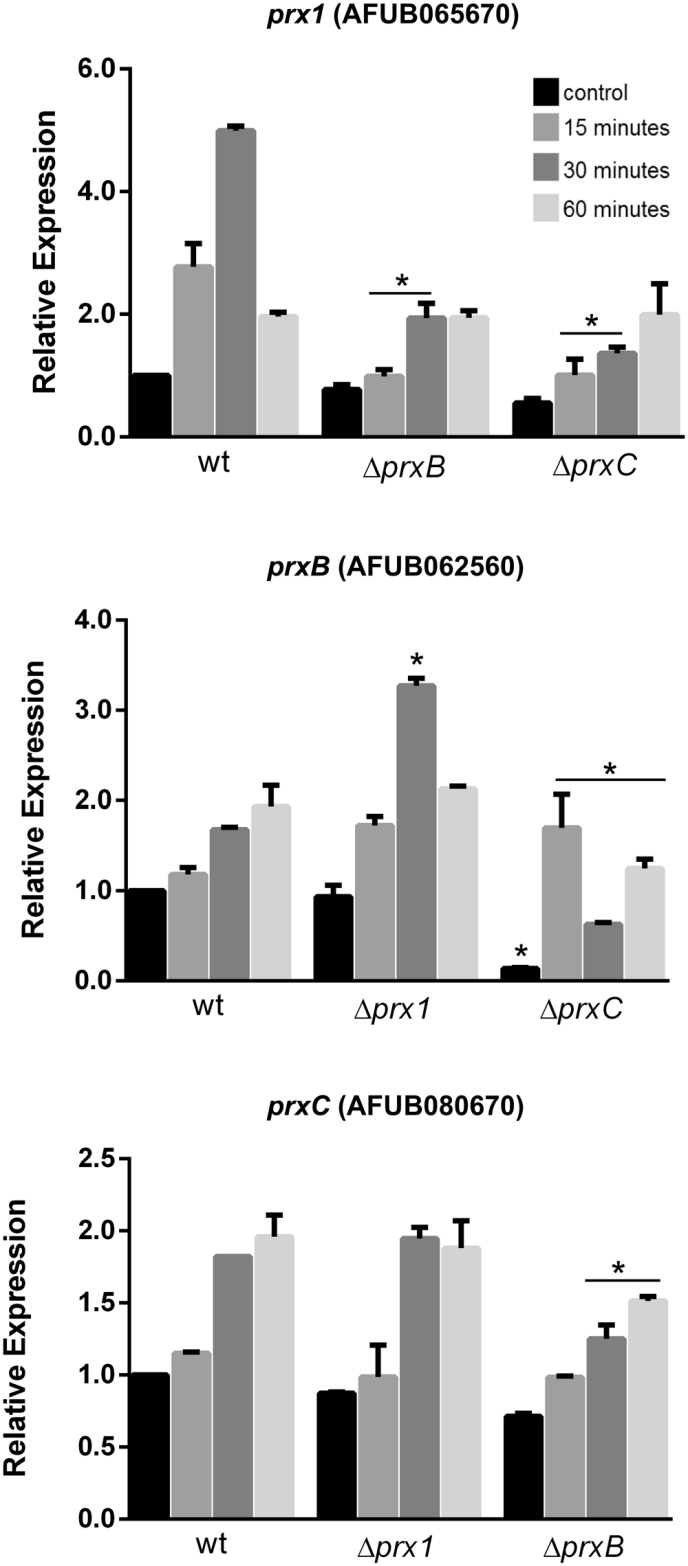
The transcriptional responses of Prxs in the presence of paraquat. Wild-type, Δ*prx1*, Δ*prxB* and Δ*prxC* mutant strains were grown in MM for 24 h and subjected to treatment with 10 mM of paraquat for 15, 30 and 60 minutes while the control was left untreated. The mRNA abundance for each gene was assessed by RT-qPCR and normalized to β-tubulin. The fold increase in each strain represents the normalized mRNA abundance relative to the wild-type. The data represent the average value of at least three biological replicates, each of which was repeated in duplicate in the same run, Bar = SD, *p ≤ 0.05 (one-way ANOVA).

To gain insights on the physiological roles of *A*. *fumigatus* 1-Cys Prxs, the expression patterns of their respective genes were characterized. Paraquat was chosen as the oxidant molecule since the single deletion mutants displayed increased sensitivity to this drug (Fig. 4). As a general trend, *prx1*, *prxB* and *prxC* genes were all induced by paraquat in a time dependent manner. However, there are considerable differences among them, such as the low induction of *prxC* (approximately 1.9-fold) in comparison to *prx1* (approximately 5.0-fold) (Fig. 6). Expressions of 1-Cys Prx genes in the mutant strains were also investigated and compensatory effects were observed only in few conditions. For instance, we observed an increase in *prxB* mRNA abundance 30 minutes after paraquat exposure in the Δ*prx1* strain. In contrast, the expression of *prxC* is comparable in the Δ*prx1* and wild-type strains.

To further analyze compensatory mechanisms, the expression of other antioxidant genes was evaluated, such as the catalases and superoxide dismutases^39,40^. Furthermore, we analyzed the expression of the transcription factor *yap1* required for oxidative stress tolerance^30^ and the putative mitochondrial cytochrome-c involved in the response to oxidative stress (yeast *CCP1* homolog)^30^. As expected, all the genes tested showed significant up-regulation after paraquat exposure (Supplementary Fig. S9). Since these antioxidant genes were still induced in the 1-Cys Prx deletion strains, no evidence for compensatory effects was obtained.

**Figure 7:**
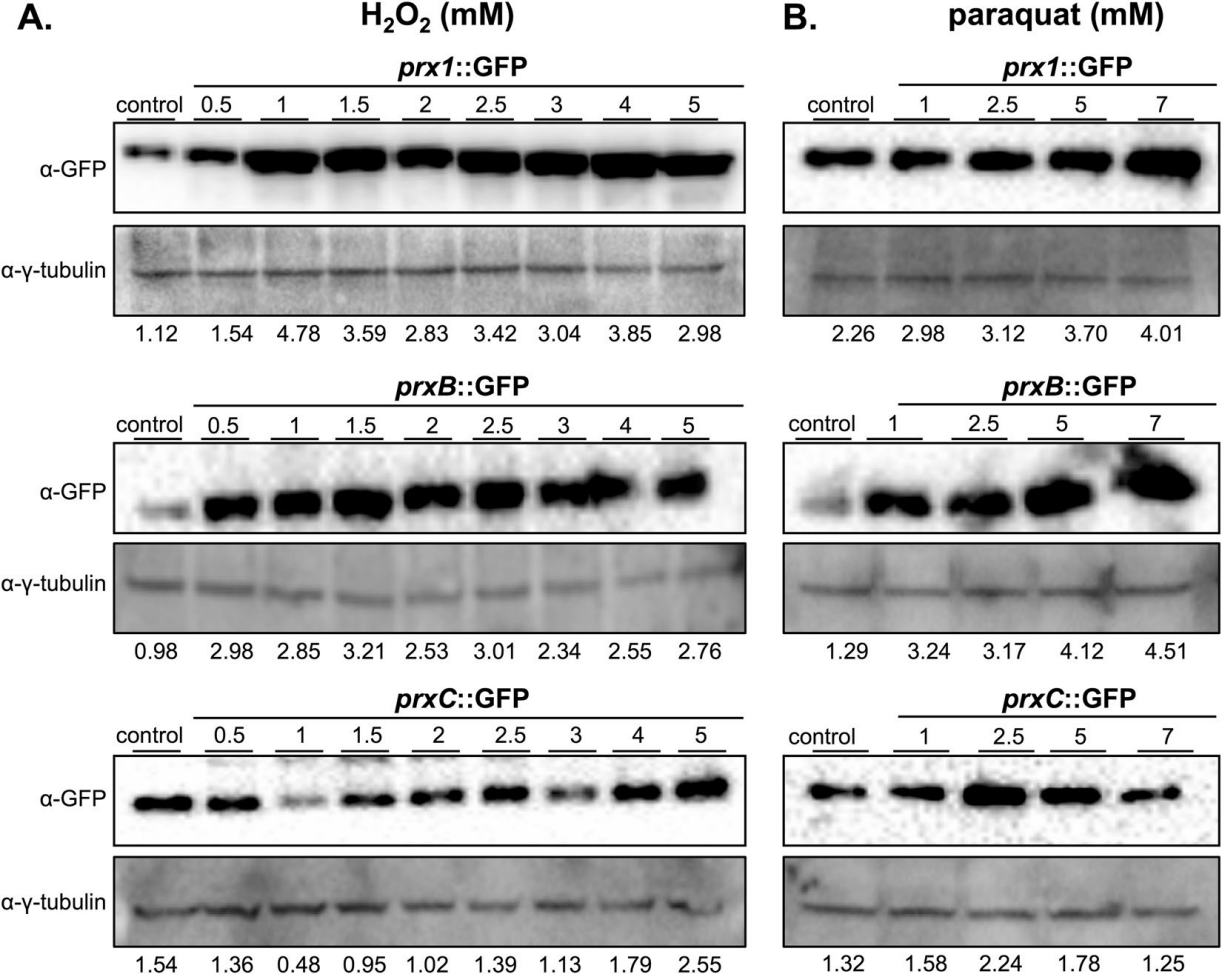
Prx1, PrxB and PrxC expression upon oxidative challenge. Prx1::GFP, PrxB::GFP and PrxC::GFP strains were grown for 24 hours in MM and subsequently exposed to increasing concentrations of H_2_O_2_ (**A**) or paraquat (**B**). The total amount of each protein was detected using an anti-GFP antibody. The anti-γ-tubulin antibody was used as a loading control. Densitometry analysis of Western blot signals is shown on the bottom of each image and represents the Prx/γ-tubulin intensity ratio. The experiments were done in triplicate and a representative experiment is shown. In the figure are reported the cropped gels/blots obtained by each protein evaluation. All gels were run in the same experimental conditions. Full-length blots are presented in Supplementary Figure S10.

The expression of 1-Cys Prxs was also evaluated at the protein level by Western blot. The detected bands migrated as predicted by their aminoacid sequences fused with GFP (Fig. 7). The expression of Prx proteins increased in response to both H_2_O_2_ and paraquat, but following distinct patterns. Expression levels of Prx1 and PrxB progressively increased. Interestingly, PrxC abundance in non-stressing conditions was higher than PrxB, but PrxB was highly inducible. Indeed, the expression of PrxC follows a constitutive pattern, although in the case of paraquat, induction was observed for treatments at 2.5 mM concentration.

### Prx1 is important for survival upon electron transport chain dysfunction

**Figure 8:**
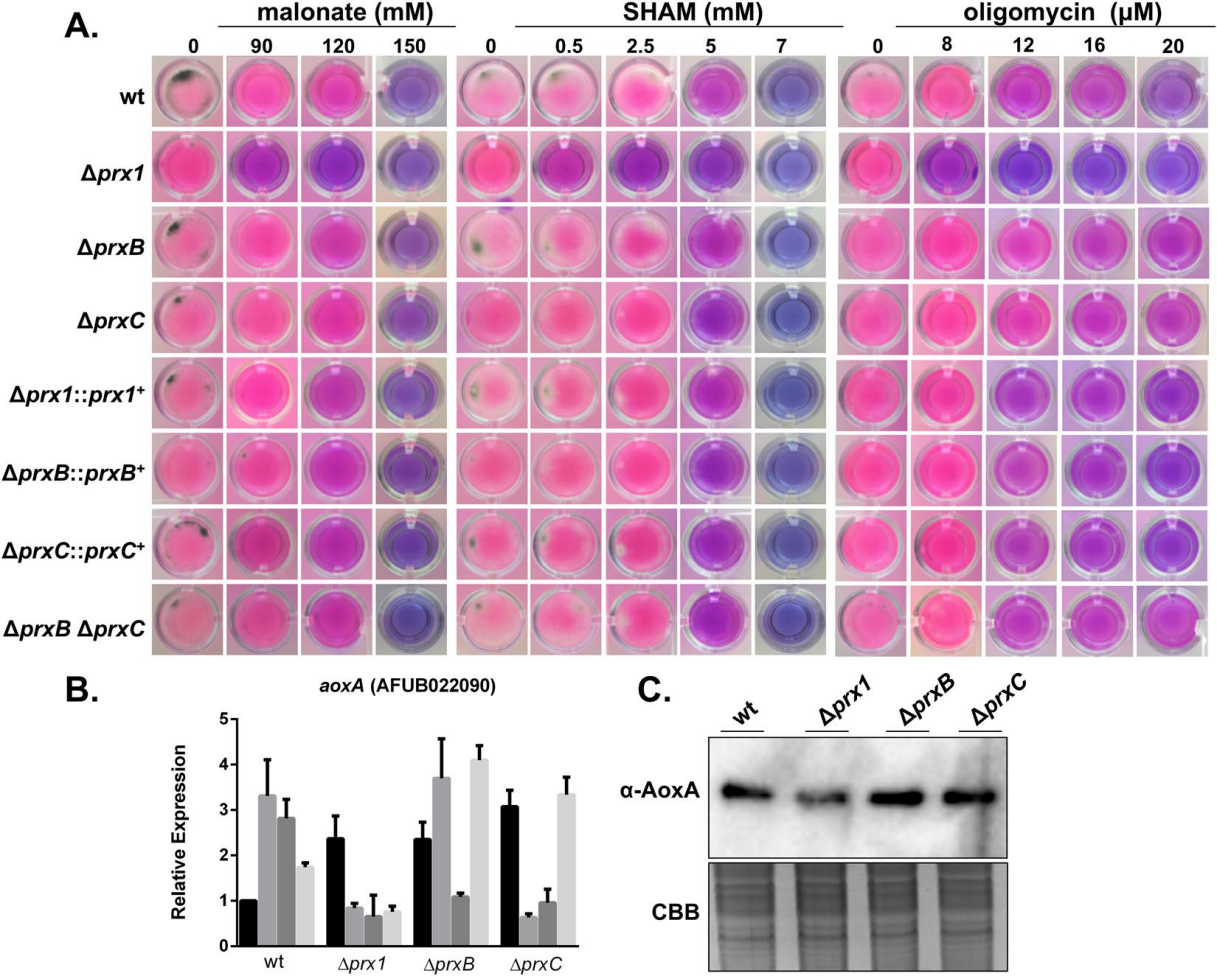
*A*. *fumigatus* Prx deletion mutants are sensitive to pharmacological inhibitors of the electron transport chain. (**A**) Viability of the germlings of the wild-type and Prx mutants in the presence of malonate, SHAM and oligomycin. Viability is shown by the indicator Alamar blue. Plates were incubated at 37 °C for 48 h and then photographed. A bluish color indicates decreased mitochondrial activity and therefore, lower viability. (**B**) Expression analysis of *aoxA* in the Prx null mutants in the presence of paraquat. The fold increase represents the normalized mRNA abundance relative to the wild-type strain. The data represent the average value of three biological replicates, Bar = SD, *p ≤ 0.05. (**C**) Western blot for AoxA expression. Anti-AoxA was used to detect the total amount of AoxA (40.8 kDa) in the mitochondrial extract. A Coomassie Brilliant Blue (CBB) stained gel was used as a loading control for the mitochondrial enrichment. Experiments were run in triplicate with similar results. Image was cropped from that shown in Supplementary Figure S13.

The role of the mitochondria and ROS in human fungal pathogenesis and drug resistance is an important area of investigation^41–43^. Therefore, we examined the effects of pharmacological inhibitors of the electron transport chain on the viability of the 1-Cys Prx deficient strains. In the presence of varying concentrations of rotenone, antimycin A and the uncoupler FCCP [carbonyl cyanide 4-trifluoromethoxy) phenylhydrazone], all three mutant strains presented sensitivity similar to wild-type cells (Supplementary Fig. S11). In contrast, the Δ*prx1* mutant displayed impaired tolerance to malonate, oligomycin and SHAM (salicylhydroxamic acid), which inhibits complex II, *F*oF1-ATP synthase and the alternative oxidase (AOX), respectively (Fig. 8A and Supplementary Fig. S12), indicating an important role of Prx1 in cell adaptation under respiratory deficiency. Again, this phenotype was not observed in the double mutant Δ*prxB* Δ*prxC*, further suggesting a prominent function of *prx1*.

AOX plays a major role in limiting mitochondrial ROS formation and consequently oxidative stress in plants^44,45^ and in *A*. *fumigatus*^41,46^. Therefore, we investigated if deletion of 1-Cys Prx genes could increase the expression of AOX from *A*. *fumigatus* (AoxA). Indeed, *aoxA* mRNA levels were twice to three times higher in the Δ*prxB* and Δ*prxC* mutants compared with the wild-type strain, in non-stressing condition (Fig. 8B). In contrast, only in the Δ*prx1* background, the *aoxA* gene was not induced by paraquat, suggesting a possible genetic interaction between Prx1 and AoxA. The lower levels of AoxA protein in the mitochondrial fractions found only for the Δ*prx1* mutant (when compared to the wild-type strain) further support this finding (Fig. 8C). It is possible that another factor is involved in the Prx1-AoxA interaction, as this Cys-based peroxidase is cytosolic and AoxA is mitochondrial.

### The MAP kinase SakA and the Yap1 transcription factor mediate transcriptional regulation of 1-Cys Prxs

**Figure 9:**
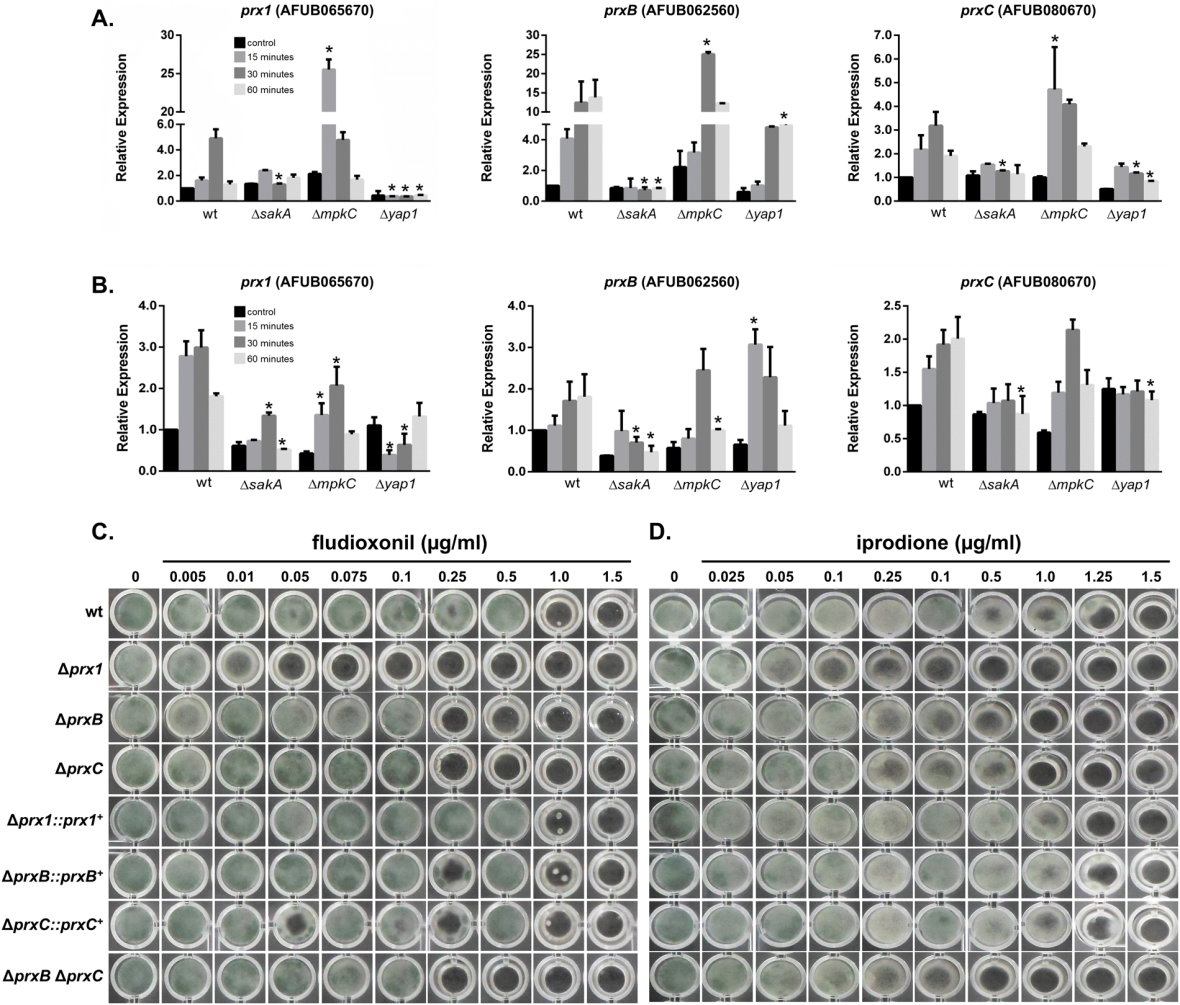
Expression of *A*. *fumigatus* Prxs is regulated by *sakA*^HOG1^ and *yap1*^YAP1^ in the presence of paraquat and fludioxonil. (**A**,**B**) mRNA abundance of *prx1*, *prxB* and *prxC* is impacted by the MAP kinase SakA while the expression of *prx1* and *prxC* is regulated by the Yap1 transcription factor. Wild-type, Δ*sakA*, Δ*mpkC* and Δ*yap1* mutant strains were grown in MM for 24 h and subjected to treatment with fludioxonil (1 µg/ml; A) or paraquat (10 mM; B) for 15, 30 or 60 minutes. The control was left untreated. The mRNA abundance for each gene was assessed by RT-qPCR and normalized to β-tubulin. The data represent the average value of three biological replicates, each of which was repeated in duplicate in the same run, Bar = SD, *p ≤ 0.05 (one-way ANOVA). (**C**,**D**) The Prx deletion mutants are more sensitive to fludioxonil and iprodione. 1 × 10^4^ conidia of each strain were grown on MM containing increasing concentrations of fungicides. The cultures were grown for 72 h at 37 °C. The results are representative of four independent experiments.

Since the expression levels of *A*. *fumigatus* 1-Cys Prxs increased upon oxidative challenge (Figs 6–7), we investigated other stressors of economic interest such as iprodione and fludioxonil, two crop fungicides. The mechanism of action of these compounds involves the hyperactivation of the HOG (High Osmolarity Glycerol) pathway and in *A*. *fumigatus*, SakA^HOG1^ and MpkC, two MAP kinases of the HOG pathway, are also involved in iprodione and fludioxonil resistance^47–51^. Here, we found that the expression of the three 1-Cys Prxs genes were up-regulated by fludioxonil in the wild-type strain; *prxB* achieving very high levels of induction by this drug (Fig. 9A). The inactivation of *sakA*, but not *mpkC*, significantly attenuated the transcriptional responses for all three Prxs to fludioxonil and paraquat (*p* ≤ 0.05; Fig. 9A,B). Noteworthy, the expression of the three 1-Cys Prxs, especially the *prx1* gene, were even higher in the Δ*mpkC* mutant background in the presence of fludioxonil. Moreover, Δ*prx1* was the most sensitive mutant to fludioxonil and iprodione, although Δ*prxB* and Δ*prxC* were also less tolerant than the wild-type strain to these fungicides (Fig. 9C,D). Interestingly, there was an epistatic interaction between *prxB* and *prxC* for the tolerance to fludioxonil (Fig. 9C,D). The levels of SakA phosphorylation increased in the time course experiment of fludioxonil treatment in the wild-type strain (Supplementary Fig. S14). Further increase in SakA phosphorylation upon fludioxonil treatment was observed only in the Δ*prx1* background after 15 minutes of exposure, which again suggests a leading role of Prx1 in fludioxonil and ROS tolerance. Collectively, these results indicate that SakA/MpkC are important for 1-Cys Prx regulation in *A*. *fumigatus*.

Yap1 is a transcription factor described as a primary determinant in the antioxidant response in yeast (reviewed in^52^). In *A*. *fumigatus*, the close homolog *yap1* is also involved in the response to oxidative challenge, but it is not required for virulence^30^. Therefore, we analyzed whether the expression of *A*. *fumigatus* 1-Cys Prx genes could also occur in a Yap1-dependent manner. The transcriptional induction of *prxC* and especially of *prx1* by paraquat and fludioxonil were significantly reduced in the Δ*yap1* and Δ*sakA* strains (Fig. 9A,B). In contrast, the expression of *prxB* was only partially regulated by Yap1.

### The Δ*prx1* strain has attenuated virulence in a neutropenic murine infection model

**Figure 10:**
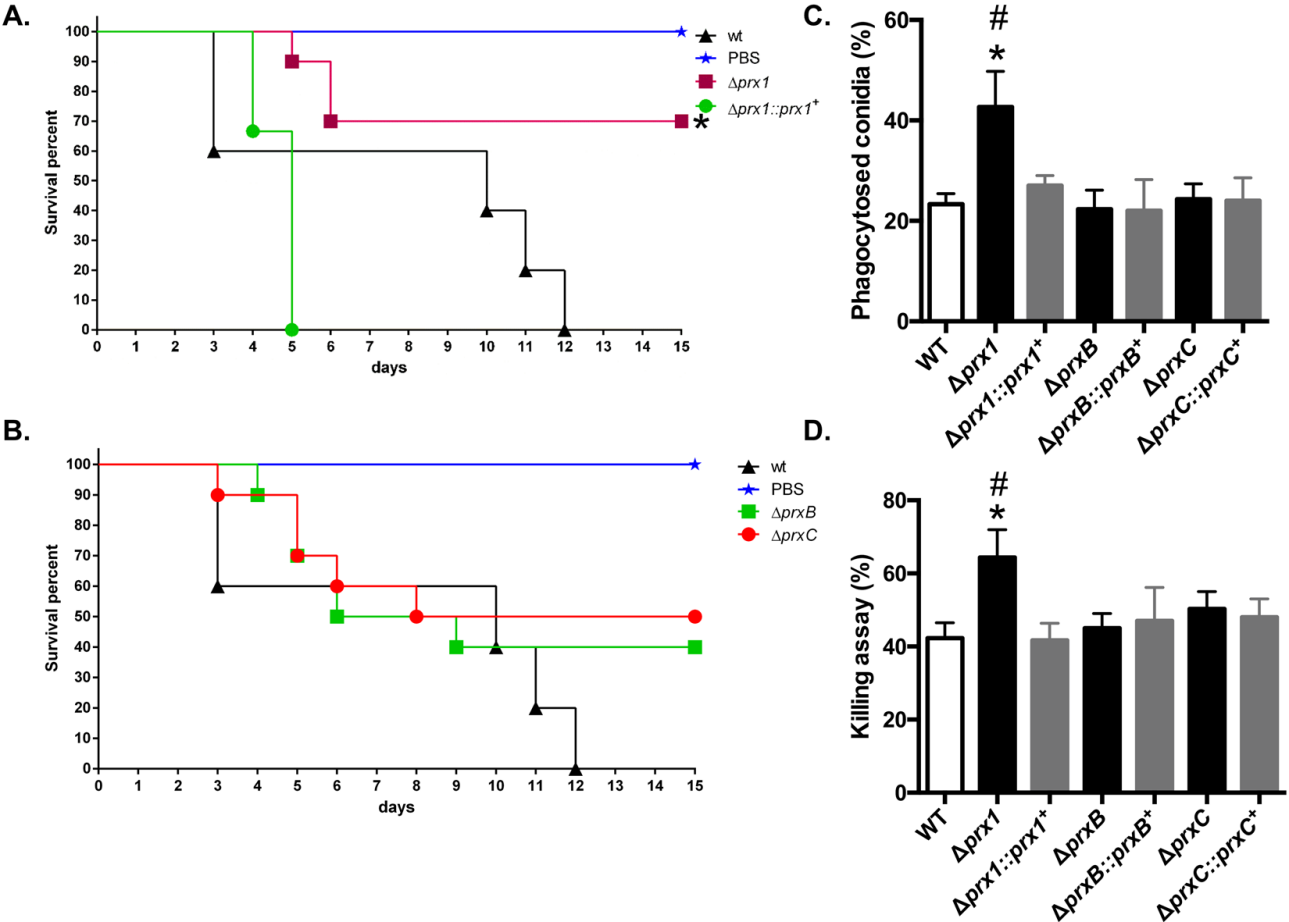
*A*. *fumigatus* Prx1, but not PrxB or PrxC contributes to virulence in a mouse model of invasive pulmonary aspergillosis. (**A**,**B**) Comparative analysis of wild-type and 1-Cys Prx mutant strains in a neutropenic murine model. No mock infected animals perished in either experiment. Survival of the mutant strain was compared to that of the wild-type and complemented strain. Infection experiments were repeated two times with similar results. Survival curves between the Δ*prx1* mutant and wild-type strain and mutant and reconstituted strains were statistically significant (*p ≤ 0.0132 and *p* ≤ 0.0074, respectively). No significant difference was observed between the wild-type and Δ*prxB* or Δ*prxC* deletion strains. Phagocytosis index (**C**) and killing activity (**D**) is increased in the *A*. *fumigatus* Δ*prx1* mutant strain. The results are expressed as means ± SD for three independent experiments. *p ≤ 0.05 compared to wild-type; ^#^*p* ≤ 0.05 compared to the complemented strain.

The involvement of the three 1-Cys Prxs in the pathogenicity of *A*. *fumigatus* was evaluated in a neutropenic murine model of invasive pulmonary aspergillosis. The Δ*prx1* infection resulted in a significant decrease in mortality rate, in a condition where wild-type infection resulted in 100% mortality 12 days post-infection (Fig. 10A). In contrast, no significant differences among the wild-type, Δ*prxB* and Δ*prxC* were observed in our experimental conditions (Fig. 10B). Virulence was fully restored in the Δ*prx1::prx1*^+^ complemented strain.

In addition, we asked whether these mutations would affect the host immune response. To investigate whether the three 1-Cys Prxs could influence the internalizing and fungicidal activity of macrophages, we compared phagocytosis index and killing levels of Bone Marrow Derived Macrophages (BMDMs) among the wild-type, mutants and complemented strains. Corroborating the survival experiments, both the phagocytosis index (Fig. 10C) and the killing levels (Fig. 10D) were significantly increased in Δ*prx1* strain when compared to wild-type strain and complemented strains. Again, no significant differences were verified in these parameters for the Δ*prxB* and Δ*prxC* mutants. We also measured the levels of TNF-α, IL-1β, and IL-12p40 from BMDMs after co-incubation with *A*. *fumigatus* conidia (Supplementary Fig. S15), but no significant differences were verified. Overall, these experimental observations suggest that Prx1 is part of the virulence arsenal of *A*. *fumigatus* and highlight the importance of 1-Cys Prxs in host-pathogen interactions.

## Discussion

Tolerance to oxidative challenge is one of the most recognized determinants of virulence in several pathogens. Although several antioxidant genes and transcriptional regulators have been characterized in *A*. *fumigatus*^30,39,40,53^, the exact contributions of these proteins in the cellular response to ROS remain elusive. This study, therefore, aimed to gain insights on how *A*. *fumigatus* antioxidant defenses are impacted by the peroxidase activity of 1-Cys Prxs.

We described here that Prx1, PrxB and PrxC hold the conserved PVC_P_TTE motif of the 1-Cys Prxs (Prx6 subfamily), as well as a fully conserved Arg residue (Fig. 1). These features are related with extremely high efficiency of Prx1 and PrxC to reduce H_2_O_2_ (rate constants in the 10^7^ M^−1^.s^−1^ range, Fig. 2). In contrast, atypical 2-Cys Aspf3 from *A*. *fumigatus* presented rate constant several orders of magnitude lower (10^4^ M^−1^.s^−1^ range), but it should be considered that this parameter was obtained by steady state kinetics (*kcat*/Km), using heterologous proteins from *E*. *coli* to turn over the enzyme^14^.

Nevertheless, Prx1 and PrxC seem to play a major role in H_2_O_2_ metabolism as these enzymes are highly reactive (Fig. 2) and also abundant (Figs 5–7). Accordingly, we observed that the Prx null mutants showed decreased tolerance to paraquat and menadione, two compounds that undergo redox cycling, generating a flux of H_2_O_2_ (Fig. 4). Unfortunately, we could not identify the nature of Prx1 and PrxC reducing agents, which is also the case for several others 1-Cys Prx. As other Prx enzymes, Prx1 and PrxC probably display a ping-pong mechanism and the determination of true K_M_ and *k*_*cat*_ parameters required the determination of rates at distinct concentrations of the reducing and oxidizing substrates.

Factors such as the regulation of gene expression, sub-cellular location and substrate affinity should determine specific roles for each 1-Cys Prx in the cellular response to H_2_O_2_. Nevertheless, in most of the conditions analyzed herein, Prx1 appears to play major roles in the response of *A*. *fumigatus* to oxidative challenge. Noteworthy, recently we developed a device to electrochemically monitor the degradation of H_2_O_2_ in the microenvironment of *A*. *fumigatus* and indeed the efficiency of H_2_O_2_ degradation for the Δ*prx1* mutant was much lower (8%), in comparison to the wild-type strain (71%)^54^.

The finding that *A*. *fumigatus* contains no typical 2-Cys Prx and three 1-Cys Prx enzymes is very unusual, since the majority of organisms possess only one functional copy of 1-Cys Prx. Therefore, we identified their sub-cellular localization to gain insights into their individual roles. In a previous proteomic study of both crude mycelial and mitochondrial protein extracts, Prx1 was detected only in the mycelial extract, PrxC was detected in both preparations, while no information was recovered for PrxB^55^. These results were confirmed here as we further advanced in determining the specific sub-cellular localization of PrxB and PrxC compartmentalization (Fig. 5).

The mitochondrial repertoire of antioxidant enzymes is quite diverse, but generally Prx enzymes play central roles in the removal of H_2_O_2_ in this organelle. For instance, mitochondria of *S*. *cerevisiae* contains only one 1-Cys Prx^56^ that is located in both matrix and intermembrane space^56^, whereas mammalian organisms contain two 2-Cys Prx (Prx3 and Prx5). Noteworthy, human Prx3 decomposes 90% of the H_2_O_2_ generated within the mitochondrial matrix^21,57,58^. N-termini cleavable leader sequences of a preprotein synthesized by cytosolic ribosomes is the classical type of mitochondrial targeting signals, as we observed for yeast Prx1 and *A*. *fumigatus* PrxC. However, additional non-cleavable targeting and sorting signals, which are located in the mature regions of mitochondrial proteins, have also been described^59–61^. This could be the case of *A*. *fumigatus* PrxB since we could not detect a canonical cleavable N-terminal presequence for this protein.

ROS production derived from the leaking of electrons from the mitochondrial transport chain is a well-established phenomenon^38,62,63^. However, these mechanisms are still scarcely understood in fungi, but there are evidences that the canonical mitochondrial respiratory pathway via cytochrome c or cyanide-resistant respiration (via AOX) impact fungal virulence and the host immune response^41,46,64–66^. Intriguingly, cytosolic Prx1 is involved in the maintenance of mitochondrial homeostasis (Fig. 8). AOX is another system involved in the decreased accumulation of mitochondrial ROS in the response of plant and fungal cells to oxidative challenge^41,42,44,67^. Curiously, we observed decreased levels of AoxA in the *prx1* deletion mutant (Fig. 8B,C) what can partially accounts for the more pronounced cell susceptibility to oxidants in this mutant (Figs 4 and 8). A potential reason for this phenotype is if AoxA is depleted, H_2_O_2_ could accumulate in mitochondria and then diffuses to the cytosol^63^. Thus, AoxA would act in parallel with Prx to counteract oxidative stress. Cytosolic Prx is important for Yap1 activation through nuclear localization in yeast^68,69^. Loss of Prx1 in *A*. *fumigatus* might impact the general response to oxidative challenge. So, in the absence of *prx1*, response of H_2_O_2_ would be decreased and levels of this oxidant should be higher in deletion mutant for *aoxA*. Confirmation of this hypothesis awaits further experimentation.

Unlike the core components of fungal respiration, the fungal AOX appears to have complex mechanisms for regulation of expression and enzyme activity which are remarkably different from plants^42,67^. Thus, a further understanding of how AOX and 1-Cys Prx activities are regulated across fungi can yield insights about the consequences for the fungal cell under accumulation of hydroperoxides.

In addition, all three 1-Cys Prxs are regulated by the HOG pathway and appear to cooperate in the response of the fungi to the crop fungicides (Fig. 9). Hyperactivation of the HOG pathway is the mechanism that explains cell death in yeast and *C*. *neoformans* while perturbations in any of the three components of the HOG pathway render *C*. *neoformans* highly resistant to fludioxonil^50,51^. Similarly, Hog1 mutants in *S*. *cerevisiae* and other filamentous fungi are also resistant to these drugs^70–73^. Although in *A*. *fumigatus* mutants for both *sakA*^HOG1^ and *mpkC* were slightly more resistant to fludioxonil^49^, we observed increased activation of SakA in the Δ*prx1* mutant (Supplementary Fig. S14). Our data indicated that SakA, and not MpkC, is necessary for the expression of 1-Cys Prxs during exposure to fludioxonil. Expression of *prx1* and *prxC* is also regulated by Yap1 (Fig. 9A,B), a transcriptional regulator that is redox regulated in yeast^74^. Accordingly, here we also identified *in silico* a conserved binding motif for Yap1 in the promoter regions of the three *A*. *fumigatus* 1- Cys Prxs (Figure S16). Functional validation of these predicted binding motifs in the transcriptional activation of the 1-Cys Prxs awaits further experimentation. A previous genome-wide study also observed that PrxC is down-regulated in a Δ*yap1* mutant exposed to H_2_O_2_^30^. How the distinct signaling pathways (HOG and Yap1) interact to integrate the responses of this pathogenic fungus to stressful conditions awaits further investigation.

Importantly, we observed that Prx1 is required for *A*. *fumigatus* virulence in our mouse model (Fig. 10). Based on data presented by this study and others^14,75^, Prx-enzymes emerge playing leading roles in *A*. *fumigatus* protection against host defenses. For instance, neither a triple *sod* mutant nor individual deletion of catalases significantly causes virulence attenuation in *A*. *fumigatus*^39,40,53^. In addition, since mammalian 1-Cys Prx (Prx6) presents acidic Ca^2+^-independent phospholipase A2 activity^24,25^, is possible that such putative activity in the *A*. *fumigatus* 1-Cys Prxs might also contribute to the fitness of the pathogen inside the host. Altogether, we cannot rule out the possibility that PrxB and PrxC are also important for virulence under other experimental conditions or additional animal models. Remarkably, BMDMs stimulated with Δ*prx1* showed enhanced phagocytosis and killing levels. Although this finding is consistent with the survival experiments, the verified cytokines levels were not significant among the analyzed mutants (Supplementary Fig. S15). Probably the possible modifications in the tolerance of *A*. *fumigatus* to oxidative damage could promote an unbalance at the phagolysosome acidification during phagocytosis and killing, but were not enough to increase the recognition of the mutated fungus by dectin-1 receptor, which would promote an exacerbated production of pro-inflammatory cytokines. Reactive oxidant intermediates have been demonstrated involved in the inhibited killing of *A*. *fumigatus* without affecting the uptake rate^76^. Also, the mechanisms of conidia killing by oxidation are still elusive since the available data in the literature for killing of *A*. *fumigatus* by macrophages are very unrelated^76^.

Taken together, our studies uncovered that the three 1-Cys Prxs act together in the *A*. *fumigatus* response to oxidative challenge with Prx1 playing a major role in virulence. We argue that further characterization of additional biochemical properties of the 1-Cys Prxs, the identification of their potential interacting proteins and understanding the mechanisms whereby they are reduced *in vivo* will help us to elucidate how *A*. *fumigatus* relies on 1-Cys Prxs to cope with ROS arising from its own metabolism and from the host during the course of infection.

## Methods

### Cloning the 1-Cys Prx genes

Sequence analysis of the *A*. *fumigatus* 1-Cys Prx were performed as described in Supplementary Methods. Prx cDNA sequences were obtained from a paraquat-induced culture of CEA17 wild-type strain. RT-PCR was run using the primer sets described in Supplementary Table S1. The PCR products were cloned into the pTZ57R/T easy vector (Thermo Scientific) and subcloned into the pET15b expression vector at the *Nde*I and *Bam*HI sites using standard protocols^77^. The pET15b/*prx1*, pET15b/*prxB* and pET15b/*prxC* clones were validated by fully sequencing.

### Protein expression and purification

Single colonies of *E*. *coli* BL21(DE3) harboring pET15b/*prx1* or pET15b/*prxC* were cultured in LB (100 μg/ml ampicillin) and grown for 16 hours (37 °C) in an orbital shaker (250 rpm). The culture was diluted in 1 L of LB (100 μg/ml ampicillin) and cultured at 37 °C until the OD_600_ reached 0.6–0.8. IPTG was then added to a final concentration of 0.3 mM and the culture was incubated at 37 °C for an additional 3 h (250 rpm). Cells were harvested by centrifugation, and the pellet was washed and suspended in the start buffer (20 mM sodium phosphate buffer, pH 7.4; 500 mM NaCl; 20 mM imidazole; 625 μM PMSF). Twenty cycles of 15 s of sonication (30% amplitude) following 40 s in ice were applied to cell suspensions. Cell extracts were kept on ice during streptomycin sulfate 1% treatment for 15 min and the suspension was centrifuged at 15,000 rpm for 45 min to remove nucleic acid precipitates and the insoluble fraction. Finally, cell extracts were filtered and purified by immobilized metal ion affinity chromatography (IMAC; HiTrap column GE Healthcare) using ÄKTA FPLC (GE Healthcare).

The conditions of protein purification were optimized using the gradient procedure for imidazole concentration as described by the manufacturer. Imidazole was removed from purified proteins by gel filtration using a PD10 column (GE Healthcare). Purified proteins were stocked in 20 mM sodium phosphate buffer (pH 7.4) containing 500 mM NaCl. Recombinant protein concentrations were determined spectrophotometrically at 280 nm. The extinction coefficients for reduced Prx1 (ε_280_ = 20,970 M^−1^.cm^−1^) and PrxC (ε_280_ = 22460 M^−1^.cm^−1^) were determined using the ProtParam tool (http://www.expasy.ch/tools/protparam.html).

### Determination of the peroxidase activity by competitive kinetics

The second-order rate constant for the reaction between Prx1 and PrxC with H_2_O_2_ was determined by competition with horseradish peroxidase (HRP)^33,78^. The Prxs were first reduced with 20 mM DTT during 16 hours at 4 °C and desalted twice using a PD10 column. Following incubation, the amount of free thiols was determined using DTNB [5,5′-dithiobis(2-nitrobenzoic acid); D8130 Sigma-Aldrich] (10 DTNB: 1 protein) under denaturing conditions (ε_412_ = 13,600 M^−1^.cm^−1^) in order to obtain the percentage of reduced protein, which was kept at above 90%. The concentration of HRP (P6782 Sigma-Aldrich) was determined spectrophotometrically using ε_403_ = 102,000 M^−1^.cm^−1^. In reaction mixtures containing 0.1 M sodium phosphate buffer (pH 7.4), 0.1 mM DTPA (diethylenetriaminepentaacetic acid, D6518 Sigma-Aldrich), 8.0 μM HRP and various concentrations of pre-reduced Prx (2–16 μM), H_2_O_2_ was added to a final concentration of 4.0 μM at 37 °C. The extent of the conversion to compound I was determined by measuring the absorbance at 403 nm before and after 30 seconds of adding H_2_O_2_. Results shown are mean ± SD obtained from three independent replicates.

### Cell fractionation

For the extraction of mitochondrial proteins, mitochondria were isolated from *A*. *fumigatus* cells based on the previous methodology^55^. Mycelia (10 g wet weight) were obtained by growing 1 × 10^8^ conidia of *prx1::gfp*, *prxB::gfp* or *prxC::gfp* strains for 16 h at 37 °C in 100 ml of YG. Cells were harvested, frozen in liquid nitrogen and subjected to cell fractionation procedures. 20 µg of protein from each sample were loaded on 12% SDS-PAGE and electroblotted to PVDF (polyvinylidene difluoride) membranes, which were then analyzed by Western blot using anti-GFP antibody.

### Strains and media

The *A*. *fumigatus* strains used in this study are described in Supplementary Table S2. Strains were grown in either complete medium (YG; 2% [wt/vol] glucose, 0.5% [wt/vol] yeast extract, trace elements) or minimal medium (MM; 1% [wt/vol] glucose, 1 × original high-nitrate salts, trace elements, pH 6.5). Trace elements, vitamins, and nitrate salt compositions were as described previously^79,80^. Solid YG and MM were the same as described above except that 2% (wt/vol) agar was added. When necessary, uridine and uracil (1.2 g/liter) were added. For all the phenotypic and expression analyses, MM was used to avoid the side reactions of peroxides with extracellular components present in a rich medium^81,82^. To induce oxidative stress, 1 × 10^7^ conidia from wild-type and mutant strains were incubated in 50 ml of liquid MM for 24 hours, at 37 °C. Following incubation, strains were challenged to increasing concentrations of H_2_O_2_ or paraquat for 30 min. Alternatively, paraquat (10 mM) or fludioxonil (1 µg/ml) was added to the cultures, and they were incubated for an additional 15, 30 or 60 min. The control was left untreated. Mycelia were collected and stored as described previously^83^.

### Generation of 1-Cys Prx mutant strains and Southern blot analyses

The gene replacement cassettes used in this study were constructed by *in vivo* recombination in *S*. *cerevisiae* as reported previously^80^ and are described in detail in the Supplementary Methods. Primers used for each mutant construction are described in Supplementary Table S3.

### Susceptibility assays against oxidative damaging agents and antifungals

To monitor growth of the mutant strains under drugs that cause oxidative stress 1 × 10^4^ conidia of each strain were grown in 1 ml of liquid MM in 24-well plates that were supplemented with varying concentrations of paraquat, menadione, iprodione and fludioxonil. The plates were incubated for 72 hours at 37 °C and photographed. The same assay was used to test the tolerance to pharmacological inhibitors of the electron transport chain, however using 1 × 10^4^ conidia of each strain grown in 200 µl of liquid MM (96-well plates) that were supplemented with varying concentrations of each compound. In these experiments, we used 10% Alamar blue (Thermo Scientific) as viability indicator, according to the procedures described previously^84^. The plates were incubated for 72 hours at 37 °C and photographed.

To generate the survival curves of the wild-type and mutant strains in the presence of oxidants (paraquat and menadione) and the electron transport chain inhibitors (malonate, SHAM and oligomycin), 1 × 10^4^ conidia of each strain were grown in 200 µl of liquid MM in black 96-well plates (CellStar 650086). Fluorescence intensities (λ = 590 nm) of treated and untreated samples for each strain were obtained using a Spectra Max SpectraMax i3 (Molecular Devices) after 24, 48 and 72 hours of incubation at 37 °C and used to calculate the percentage of growth, according to the manufacturer’s protocol (Thermo Fisher #DAL1100).

Sensitivity to the oxidative damage generated by H_2_O_2_ was evaluated by an inhibition zone assay on MM agar plates, as described previously^30^. Alternatively, to test the susceptibility of the strains to antifungal drugs, serial dilutions of wild-type and null mutant conidia were spotted onto agar plates.

### RNA extraction and gene expression analysis

The total RNA obtained upon cell treatments with paraquat or fludioxonil was extracted and processed for cDNA synthesis as previously described^83^. The primers for the individual genes are listed in Supplementary Table S4. Three independent biological replicates were used and the relative fold change in mRNA quantity was calculated using comparative cycle threshold (Ct) (ΔΔCt) analysis^85^. All values were normalized to the expression of the *A*. *fumigatus tubA* gene. Statistical analysis was performed using one-way ANOVA with Tukey’s *post hoc* test to assess differences in the mutant strains compared to the same growth condition in the wild-type strain (*p* ≤ 0.05).

### Immunoblot analysis

The GFP-tagged Prx strains either non-stressed or exposed to H_2_O_2_ or paraquat were used to assess the sub-cellular localization and protein abundance. Protein extraction and quantification was performed as described previously^83^. 20 µg of protein from each sample were resolved in a 12% (w/v) SDS-PAGE and transferred to PVDF membranes (BioRad). Detailed information about the conditions used for anti GFP (G1544; Sigma), Anti-γ-tubulin (yN-20; Santa Cruz Biotechnology), Anti *S*. *cerevisiae* Pgk1 (NE130/7 S; Nordic-Immunology), anti- phospho-p38 MAPK or anti-p38 MAPK (9211 and 9212, respectively; Cell Signaling Technologies) and anti- alternative oxidase (AoxA) (a gift from Drs. Sergio Akira Uyemura and Taisa Magnani Dinamarco, USP, Brazil; unpublished) are described in Supplementary Methods.

### Microscopy

Prx1::GFP, PrxB::GFP and PrxC::GFP conidia were cultivated on 35 mm Glass-Botton dishes (MatTek Corporation) in 2 ml of MM for 16 h at 30 °C. For co-localization experiments, germlings were co-stained with MitoTracker DeepRed (Thermo Scientific) as described previously^86^. Coverslips were inspected on Observer Z1 fluorescence microscope (Carl Zeiss). GFP and Mitotracker DeepRed were visualized using 38 HE and 63 HE filter sets (Carl Zeiss), respectively. DIC (differential interference contrast) and fluorescent images were captured with an AxioCam camera (Carl Zeiss) and processed using AxioVision software.

### Virulence assay

The virulence of *A*. *fumigatus* strains was analyzed using a murine model for invasive aspergillosis, as detailed previously^87^. Outbreed female mice (BALB/c strain; body weight, 20 to 22 g) were housed in vented cages containing five animals. The mice were immunosuppressed with cyclophosphamide at 150 mg/kg body weight, which was administered intraperitoneally on days -4, -1, and 2 prior to and post-infection. Hydrocortisone acetate (200 mg/kg) was injected subcutaneously on day -3. The *A*. *fumigatus* conidia that were used for inoculations were grown on solid YG medium for 2 days prior to infection. The conidia were freshly harvested in PBS and filtered using Miracloth (EMD Millipore). Conidial suspensions were spun for 5 min at 3,000 x g, washed three times with PBS, counted using a hemocytometer, and resuspended at a concentration of 5 × 10^6^ conidia/ml. Viable counts of the administered inocula were determined, following serial dilution, by plating on solid YG medium, and the conidia were grown at 37 °C. The mice (10 animals) were anesthetized by halothane inhalation and infected through intranasal instillations of 1 × 10^5^ conidia in 20 µl of PBS. For the negative control, a group of 10 mice received sterile PBS only. Mice were weighed every 24 hours starting from the day of infection and visually inspected twice daily. The statistical significance of comparative survival values was calculated using the Prism statistical analysis package by using Log-rank (Mantel-Cox method) and the Gehan-Breslow-Wilcoxon test.

### Ethics statement

This study and all protocols involving animal care were approved by the local Ethics Committee for Animal Experiments (Campus of Ribeirão Preto, Universidade de São Paulo; Permit Number: 08.1.1277.53.6). All animals were housed within individually ventilated cages and were cared for in strict accordance with the principles outlined by the Brazilian College of Animal Experimentation (Colégio Brasileiro de Experimentação Animal, COBEA) and Guiding Principles for Research Involving Animals and Human Beings, American Physiological Society. The principles that guided our studies were based on the Declaration of Animal Rights ratified by the UNESCO in January 27, 1978 (8^th^ and 14^th^ articles). All efforts were made to minimize suffering. The animals were humanely sacrificed if moribund (as defined by lethargy, dyspnea, hypothermia and weight loss). All stressed animals were sacrificed by cervical dislocation.

### Bone marrow-derived macrophages (BMDMs) preparation, phagocytosis and killing assay

All experiments with macrophages in this study were performed with BMDMs. The BMDMs preparation was performed according to^88^ with slight modifications. BMDMs were prepared from C57BL/6 adult mice femurs and tibias after flushing with RPMI medium to release bone marrow cells. These cells were cultured for 6 days in RPMI 1640 medium supplemented with 20% fetal cow serum (FCS) and 30% L-929 cell conditioned medium. Non-adherent cells were removed and the adherent cells (majority macrophages) were removed and washed twice with cold PBS. Cell concentration was determined using a Neubauer chamber.

The phagocytic assay was performed as previously described^89^ with slight modifications. In 24-well microplates at 37 °C with 5% CO_2_, 1 ml of RPMI-FCS containing 1 × 10^5^ conidia (1:5 macrophage/conidia ratio) was added and incubated for 90 min. The supernatant was removed and added 0.5 ml of 3.7% formaldehyde-PBS. The samples were washed with ultrapure water and incubated for 20 min with 495 μl of water and 5 μl of CFW (10 mg/ml). The samples were washed and mounted on slides with 50% glycerol. The images were acquired on a fluorescence microscope Eclipse E800 (Nikon Instruments), and the phagocytosis index calculated counting at least 100 conidia per sample. The experiments were repeated in triplicate.

To assess conidial killing, the phagocytic cells were obtained as described above. Subsequently, 1×10^5^ conidia (1:5 macrophage/conidia ratio) were incubated at 37 °C with 5% CO_2_ for 4 h. As positive control, conidia without BMDMs were used. The 24-well microplates were centrifuged for 10 min at 3,500 rpm, and the culture supernatant removed. Then, 100 μl of 1% Triton X-100 were added. After 10 min at room temperature, samples were removed from the microplate and serially diluted in PBS. The dilutions were plated and incubated at 37 °C for 48 h. The percentage of conidial killing was calculated by the measurement of colony forming units (CFU) numbers carried out after macrophage lysis, compared from samples incubated with macrophages to CFU numbers from those incubated without macrophages. The experiments were repeated three times, each performed in triplicate.

### Cytokine measurements

Cytokines were measured by capture enzyme-linked immunosorbent assay (ELISA) as previously described^90^ with antibody pairs purchased from BD Biosciences. Supernatants were used to quantify the levels of TNF-α, IL-1β, and IL-12p40. The amount of cytokine was determined from standard curves, using murine recombinant cytokines as standards. Data are the average of at least three independent experiments, each performed in triplicate.

### Data Availability

All data generated or analyzed during this study are included in this published article (and its Supplementary Information files).

## Electronic supplementary material

Supplementary Information
