# Supplementary material for: Analyses of the three 1-Cys Peroxiredoxins from Aspergillus fumigatus reveal that cytosolic Prx1 is central to H2O2 metabolism and virulence
Source: Sci Rep. 2018 Aug 17;8:12314. doi: 10.1038/s41598-018-30108-2 (PMC6098058; doi:10.1038/s41598-018-30108-2)
Supplement: Supplementary file 1 — Supplementary Information [file 41598_2018_30108_MOESM1_ESM.pdf]

Supplementary Information

Analyses of the three 1-Cys Peroxiredoxin from *Aspergillus fumigatus* reveal that cytosolic Prx1 is central to H_2O_2 metabolism and virulence

Marina Campos Rocha[1+], Krissia Franco de Godoy[1+], Renata Bannitz-Fernandes[2], João H. T. Marilhano Fabri[1], Mayra M. Ferrari Barbosa[1,3], Patrícia Alves de Castro[4], Fausto Almeida[5], Gustavo Henrique Goldman[4], Anderson Ferreira da Cunha[1], Luis E. Soares Netto[2], Marcos Antonio de Oliveira[6], Iran Malavazi[1]*

[1] Departamento de Genética e Evolução, Centro de Ciências Biológicas e da Saúde, Universidade Federal de São Carlos, São Carlos, SP, 13.565-905, Brazil.

[2] Departamento de Genética e Biologia Evolutiva, Instituto de Biociências, Universidade de São Paulo, SP, 05508-090, Brazil.

[3] Present address: Instituto Butantan, São Paulo, SP, 05503-900, Brazil.

[4] Departamento de Ciências Farmacêuticas, Faculdade de Ciências Farmacêuticas de Ribeirão Preto, Universidade de São Paulo, Ribeirão Preto, SP, 14.040-903, Brazil.

[5] Departamento de Bioquímica e Imunologia, Faculdade de Medicina de Ribeirão Preto, Universidade de São Paulo, Ribeirão Preto, SP, 14.040-900, Brazil.

[6] Instituto de Biociências, Universidade Estadual Paulista, Campus do Litoral Paulista, São Vicente, SP, 11.380-972, Brazil.

* imalavazi@ufscar.br

[+] these authors contributed equally to this work

Supplementary Methods

Sequence analysis of *A. fumigatus* 1-Cys Prx

The 1-Cys Prx sequences from *Homo sapiens* and *S. cerevisiae* (NCBI accession number: NP_004896.1 and NP_009489.1) were used to identify similar sequences at the AspGD (http://www.aspergillusgenome.org). These sequences identified in the *A. fumigatus* strain FGSC A1163 were then used as queries to analyze at the National Center for Biotechnology Information Genbank using the tBLASTX search tool. The selected sequences were aligned and scoring protocols were performed using the Clustal Ω alignment tool[1]. The graphical representations were performed using the Jalview software[2]. 1-Cys Prx sequences from *A. fumigatus* were analyzed using the Mitoprot II software to predict the mitochondrial localization peptide signal according to reference[3].

Generation of 1-Cys Prx mutant strains

For the deletion cassettes, fragments encompassing the 5' and 3' UTR regions of the Prx genes: Afu4g08580/AFUB_065670 (*prx1*); Afu5g15070/AFUB_062560 (*prxB*) and Afu8g07130/AFUB_080670 (*prxC*) were PCR-amplified from genomic DNA of the CEA17 strain according to Supplementary Figures S3A, S3E and S3I. The primer sequences are listed in Supplementary Table S3. The 5' and 3' Prx flanking regions contained a short sequence that was homologous to the multiple cloning site of the pRS426 plasmid (underlined letters in Supplementary Table S3). The *pyrG* gene (1,911 bp) was used as a prototrophy marker in the deletion cassettes and was amplified from the pCDA21 plasmid[4]. Deletion cassettes were generated by transforming the three independent fragments along with the *Bam*HI-*Eco*RI-cut pRS426, into the *S. cerevisiae* FGSC 9721 strain as

previously described[5]. The isolated plasmids harboring the gene replacement cassettes were used as templates to PCR-amplify the cassettes with the outermost primers (5F and 3R) indicated in Supplementary Figures S3B, S3F and S3J. All PCR amplifications were performed using Phusion Flash High-Fidelity DNA Polymerase (Thermo Scientific). The gene replacement cassettes were transformed into protoplasts of the *A. fumigatus* Δ*akuB*^{KU80} *pyrG-* according to standard protocols[5]. Genomic Southern blot genotyping confirmed single-copy deletion of the Prx genes in at least one transformant, and these clones were used for phenotypic analysis (Supplementary Figures S3C, S3G and S3K).

To complement the null mutant strains, we initially generated the strains Δ*prx1 pyrG*$^-$, Δ*prxB pyrG*$^-$ and Δ*prxC pyrG*$^-$ by the spontaneous loss of the *pyrG* prototrophy marker upon 5-fluoroorotic acid treatments. Subsequently, each Prx gene plus the 5' and 3' flanking regions (approximately 1.0 kb) were PCR-amplified using the genomic DNA from the CEA17 wild-type strain and the primers 5F_NEW and 3R_NEW (Supplementary Table S3). Protoplasts from each Prx deletion *pyrG*$^-$ strain were co-transformed with the corresponding gel-purified complementing cassette and the plasmid pCDA21 in a 10:1 ratio according to reference[6] and plated onto solid YG selective medium. Several colonies, which were able to grow under these conditions, were further analyzed by PCR to confirm the re-introduction of *prx1*, *prxB* or *prxC* as an ectopic transgene (Supplementary Figures S3D, S3H and S3L).

To construct the double mutant Δ*prxB* Δ*prxC*, the Δ*prxB* deletion cassette was PCR-amplified from the pRS426 plasmid containing the recombined cassette (primer set Afu5g15070 5F_NEW and Afu5g15070 3F_NEW) and transformed into the Δ*prxC pyrG*$^-$ recipient strain. The *prxB* gene replacement in this mutant was checked

by PCR with the primer set Afu5g15070 500 ups and Afu5g15070 3R_NEW (Supplementary Figure S3M).

To generate a translational fusion of each Prx with green fluorescent protein (GFP), substitution cassettes were constructed in which genomic sequences of *prx1*, *prxB* and *prxC* with no stop codon were cloned in-frame with the GFP gene in a C-terminal fusion (Supplementary Figure S3N, S3P and S3R). A four-residue linker consisting of Gly-Thr-Arg-Gly was inserted between the C-terminus of the Prx gene and the start codon of GFP, as described previously[7]. The GFP gene (726 bp) was PCR-amplified from the pMCB17apx plasmid by using the primers Spacer GFP FW and GFP REV pyrG. The *pyrG* gene was also used as a marker for prototrophy. The amplification of the 3' UTR region of each gene was performed with the same primers that were used in the construction of the deletion cassettes (Supplementary Table S3). A *S. cerevisiae in vivo* recombination assay was performed as previously described. The PCR-amplified fragment of each GFP fusion cassette was transformed into the *A. fumigatus* wild-type strain. Transformants were tested by PCR with primers 5F and GFP REV pyrG to confirm the gene replacements (Supplementary Figures S3O, S3Q and S3S).

DNA manipulation and Southern blot analysis

Southern blot analysis was used to show that the deletion cassette for *prx1*, *prxB* or *prxC* integrated homologously at the *A. fumigatus* genome. Genomic DNA from wild-type and mutant strains was extracted as previously described[5]. For Southern blot analysis, *Bam*HI or *Eco*RI- restricted chromosomal DNA fragments were separated on a 1% agarose gel and blotted onto Hybond N^+ nylon membranes (GE Healthcare) following standard techniques for DNA manipulation[8]. Probe

labeling for detection was performed using AlkPhos Direct Labelling and Detection System (GE Healthcare) according to the manufacturer's description. Labeled membranes were exposed to ChemiDoc XRS gel imaging system (BioRad) to generate the images.

Protein extraction and immunoblotting analysis

Polyvinylidene difluoride (PVDF) membranes obtained for each experimental condition (Bio Rad) were blocked for four hours at room temperature in 5% skimmed milk in TBST buffer (137 mM NaCl, 20 mM Tris and 0.1% Tween-20).

Anti-GFP antibody (G1544; Sigma) was used at a 1:10,000 dilution in TBST containing 3% skimmed milk. Incubation was performed at 4°C for 16 h. The primary antibody was detected with an HRP-conjugated secondary antibody raised in rabbits (A0545; Sigma). The anti-GFP antibody detected bands with the following estimated molecular weights: 51.4 kDa, 49.8 KDa and 56.4 KDa, corresponding to GFP fusions with Prx1, PrxB and PrxC, respectively.

Anti-γ-tubulin (yN-20; Santa Cruz Biotechnology) was used as the loading control in these experiments. The incubation was performed at a 1:1,000 dilution in TBST containing 3% skimmed milk 16 h at 4°C. Anti-γ-tubulin antibodies were detected with peroxidase (HRP)-conjugated secondary antibody (sc-2020; Santa Cruz Biotechnology). Anti *S. cerevisiae* Pgk1 (NE130/7S; Nordic-Immunology) was used at 1:3,000 dilution in TBST 5% skimmed milk as a control of cell fractionation. Anti-Pgk1 recognizes a single band (44.8 kDa) corresponding to *A. fumigatus* PgkA (AFUB009760) in the cytosolic crude extract. The phosphorylated and the total amount of $SakA^{HOG1}$ were examined using anti- phospho-p38 MAPK or anti-p38 MAPK (9211 and 9212, respectively; Cell Signaling Technologies) as previously

described[9]. Alternative oxidase (AFUB022090; *aoxA*) levels were detected in mitochondrial extracts using anti *A. fumigatus* AoxA polyclonal antibody raised in rabbits (a gift from Dr. Sergio Akira Uyemura and Dr. Taisa Magnani Dinamarco, USP, Brazil; unpublished).

All the primary antibody detections here were performed at room temperature with a 1:3,000 dilution of the aforementioned secondary antibody in TBST buffer for a two-hours incubation. Chemoluminescent detection was performed by using an ECL Prime Western Blot detection kit (GE HealthCare). Images were generated by exposing the PVDF membranes to the ChemiDoc XRS gel imaging system (BioRad). The images were subjected to densitometric analysis using the ImageJ software[10].

Supplementary Tables

Supplementary Table S1. Primers used in this study for cloning full-length Prxs cDNA.

Primer name	Sequence (5' → 3')
AfPrx1_F	CGGATCCCATATGGCCCAGGAACGTGC
AfPrx1_R	CAAGCTTGGATCCTTACGGCTTGACCTC
AfPrxC_F	CGGATCCCATATGGCATCCATTCTACCC
AfPrxC_R	CAAGCTTGGATCCCTAGAACTTGGTGTAG

The underlined bases represent the *Nde* I and *Bam* HI restriction sites.

Supplementary Table S2. *A. fumigatus* strains used in this study.

Name	Genotype	Reference
CEA17 (FGSC 1152[a])	*pyrG-*, *MAT1-1* (wild-type strain)	[11]
Afs35 (FGSC 1159)	*akuA::loxP* (wild-type strain)	[12]
ΔKU80 pyrG1 (FGSC A1160)	Δ*akuB; pyrG1 MAT1-1*	[13]
Δ*sakA*	*sakA::hph*; Hyg^R	[14]
Δ*mpkC*	Δ*mpkC::hph;* Hyg^R	[14]
Δ*yap1*	Δ*yap1::hph*, Hyg^R	[15]
Δ*prx1*	Δ*prx1::pyrG;* Δ*akuB*	This study
Δ*prxB*	Δ*prxB::pyrG;* Δ*akuB*	This study
Δ*prxC*	Δ*prxC::pyrG;* Δ*akuB*	This study
Δ*prx1:: prx1*$^+$	Δ*prx1:: prx1*$^+$*;* Δ*akuB*	This study
Δ*prxB:: prxB*$^+$	Δ*prxB:: prxB*$^+$*;* Δ*akuB*	This study
Δ*prxC:: prxC*$^+$	Δ*prxC:: prxB*$^+$*;* Δ*akuB*	This study
Δ*prx1 pyrG*$^-$	Δ*prx1::* Δ*akuB*	This study
Δ*prxB pyrG*$^-$	Δ*prxB::* Δ*akuB*	This study
Δ*prxC pyrG*$^-$	Δ*prxC::* Δ*akuB*	This study
prx1::gfp	*prx1::gfp;* Δ*akuB*	This study
prxB::gfp	*prxB::gfp;* Δ*akuB*	This study
prxC::gfp	*prxC::gfp;* Δ*akuB*	This study
Δ*prxB* Δ*prxC*	Δ*prxB::;* Δ*prxC::pyrG*$^+$*;* Δ*akuB*	This study

[a]FGSC: Fungal Genetics Stock Center (http://fgsc.net)

Supplementary Table S3. Primers used in this study for construction of mutant strains.

Primer name[a]	Sequence (5' → 3')[b]
Afu4g08580 5F	GTAACGCCAGGGTTTTCCCAGTCACGACGAGTCCTCGCAAGAATGCAACCA
Afu4g08580 5R	gcctcctctcagacagaattccTGGAATGGGAAGAGACAACGGA
Afu4g08580 3F	gtttgaggcgaattcgatatcTTACCTGCGCTTCACCACTCTT
Afu4g08580 3R	GCGGATAACAATTTCACACAGGAAACAGCACCCGATGTCTGCCACTTTCAT
Afu5g15070 5F	GTAACGCCAGGGTTTTCCCAGTCACGACGTGAGAGGAGAAGAGCACAGTAGGA
Afu5g15070 5R	gcctcctctcagacagaattccTGTGCTGCTTGCTTGTGGAT
Afu5g15070 3F	gtttgaggcgaattcgatatcGATGTTATGTCGAATTTCTCTTTC
Afu5g15070 3R	GCGGATAACAATTTCACACAGGAAACAGCGGTTGGATAAAACCTTGTATGCC
Afu8g07130 5F	GTAACGCCAGGGTTTTCCCAGTCACGACGTTCCGTATCTAGCCCCGC
Afu8g07130 5R	gcctcctctcagacagaattccTGTGCAGATCGACGAGAAA
Afu8g07130 3F	gtttgaggcgaattcgatatcATGCCGGTCGATACATGG
Afu8g07130 3R	GCGGATAACAATTTCACACAGGAAACAGCTCCTCCTCGGTTGTGGTG
Afu4g08580 500 ups	GAAATGCAGGTCTTTGGGG
Afu5g15070 500 ups	AGCCTTGAAAACCATTAACAATG
Afu8g07130 500 ups	CGTGTAGCTGCCACTGCC
Afu4g08580 F_NEW	AGTCCTCGCAAGAATGCAACCA
Afu4g08580 R_NEW	TCCAGCTCGTGGTACATGG
Afu5g15070 5F_NEW	CCGCTGGGTTATGCAGAC
Afu5g15070 3R_NEW	GACCGCCTTACAAAGAACCTC
Afu8g07130 5F_NEW	CATGACTCCTCTTCAAGTGCAT
Afu8g07130 3R_NEW	GACCCGAATCAAATCTCCTG
pyrG FW	GGAATTCTGTCTGAGAGGAGGC
pyrG REV	GATATCGAATTCGCCTCAAAC
Spacer GFP FW	**GGAACACGGGGA**ATGAGTAAAGGAGAAGAACTTTTCA
GFP REV pyrG	gcatcagtgcctcctctcagacagaattccTTATTTGTATAGTTCATCCATGCCATG
Afu4g08580 5F 1225	GTAACGCCAGGGTTTTCCCAGTCACGACGACCGAAAACAGCAAGTGTGG

Afu4g08580 ORF REV	agttcttctcctttactcattccccgtgttccCGGCTTGACCTCACGG
Afu5g15070 5F 1312	GTAACGCCAGGGTTTTCCCAGTCACGACGTATCAAACAGGCGCTCATTTC
Afu5g15070 ORF REV	agttcttctcctttactcattccccgtgttccATGCTGATAAAGATGGTGCCT
Afu8g07130 5F 1293	GTAACGCCAGGGTTTTCCCAGTCACGACGGCATCTTCACCGTGTGTTTCT
Afu8g07130 ORF REV	agttcttctcctttactcattccccgtgttccGAACTTGGTGTAGCGCAGGT
GFP REV_109	CATCACCTTCACCCTCTCCA

[a]For primers location refer to Supplementary Figure S3.
[b]Small letters indicate homology to the pRS426 flanking sequence according to reference[5] .
Underlined letters indicate homology to a fragment in the cassette.
Bold letters indicate the Gly-Thr-Arg-Gly linker separating the Prx gene C-terminus and GFP start codon according to reference[7].

Supplementary Table S4. RT-qPCR primers used in this study

Gene	Systematic name*	Primer name	Sequence (5' →3')
prx1	Afu4g08580	Afu4g08580 FW	CTCTACTGCCCCTGACTTCG
		Afu4g08580 REV	GGGTGGGAGAAGAGGATAGC
prxB	Afu5g15070	Afu5g15070 FW	GGGCCATATGCTGTTCAAGT
		Afu5g15070 REV	GATTGGTGCTTGGCTAGGTC
prxC	Afu8g07130	Afu8g07130 FW	CAATGCTCTACCCTGCTTCC
		Afu8g07130 REV	CAATGCCCTTCTTGTCTGCT
aoxA	Afu2g05060	aoxA FW	CACCGCGAGGTCAACCA
		aoxA REV	GGTTCGGATCCACATTGTGTT
ccp1	Afu4g09110	ccp1FW	TCCCCGACGCTTCCAA
		ccp1 REV	TCCCATGCGGTAGAAGATGTC
sod1	Afu5g09240	sod1 FW	CAAGATCACCGGCACTGTCA
		sod1 REV	AGACGGTGGTGGGAGAGTTCT
sod2	Afu4g11580	sod2 FW	GCTTCGGCTGCTCCAAGA
		sod REV	CCTTGCCGCGAGCAAA
cat1	Afu3g02270	cat1 FW	TCGGCCCCTGCAGATTC
		cat1 REV	AGCGCCCCAACAGTCTTG
cat2	Afu8g01670	cat2 FW	ACATTGCCGCGCTCAAG
		cat2 REV	GCGGTGGAGATGAAGCTTCT
yap1	Afu6g09930	yap1 FW	GGAAGCCCATCCCACAATT
		yap1 REV	TCTTTGGCGCTGCTGGTT
tubA	Afu1g10910	tubA FW	TTCCCAACAACATCCAGACC
		tubA REV	CGACGGAACATAGCAGTGAA

* *Aspergillus* genome database (http://www.aspgd.org)

Supplementary Figures

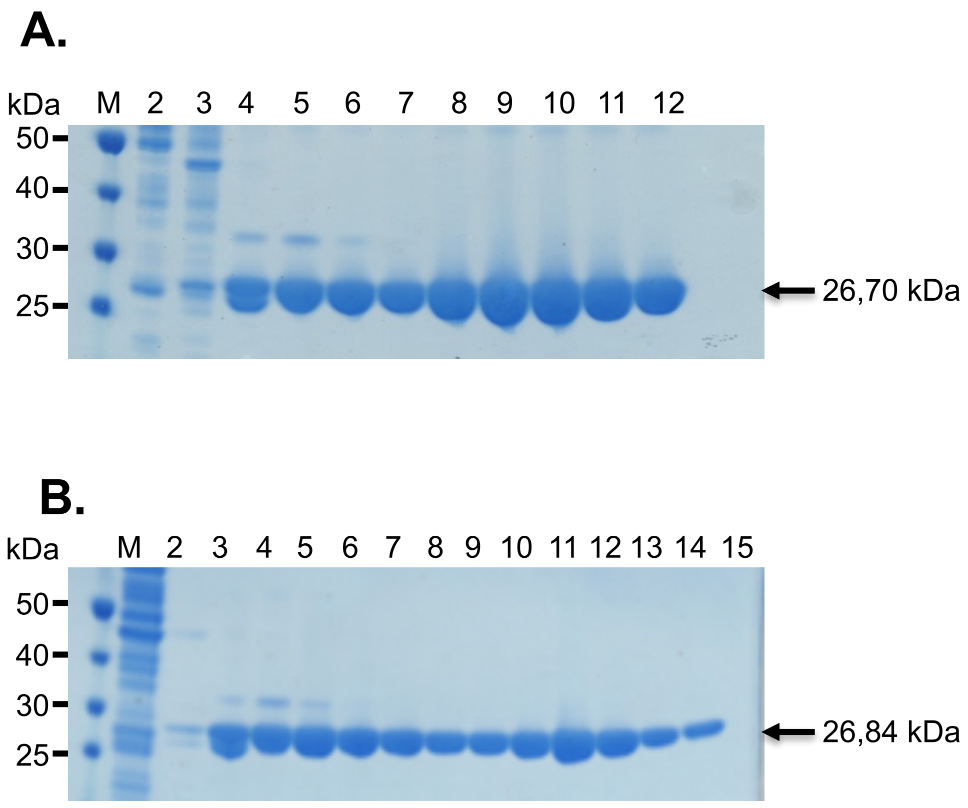

Supplementary Figure S1. Expression and purification of *A. fumigatus* recombinant 1-Cys Prxs**.** Prx1 (A) and PrxC (B) were expressed as His-tagged proteins and purified by nickel affinity chromatography (IMAC) using an imidazole gradient. For both figure panels, M is the molecular weight marker (kDa). The eluted fractions of the imidazole gradient (0 - 125 mM) were applied to the other lanes as indicated. (A) Lanes 2-7 imidazole gradient (0 - 100 mM), lanes 8-12 elution of imidazole 125 mM. (B) Lanes 2-9 imidazole gradient (0 - 100 mM), lanes 10-15 elution of imidazole 125 mM. Both samples eluted in 125 mM imidazole were concentrated, desalted and used in the assay.

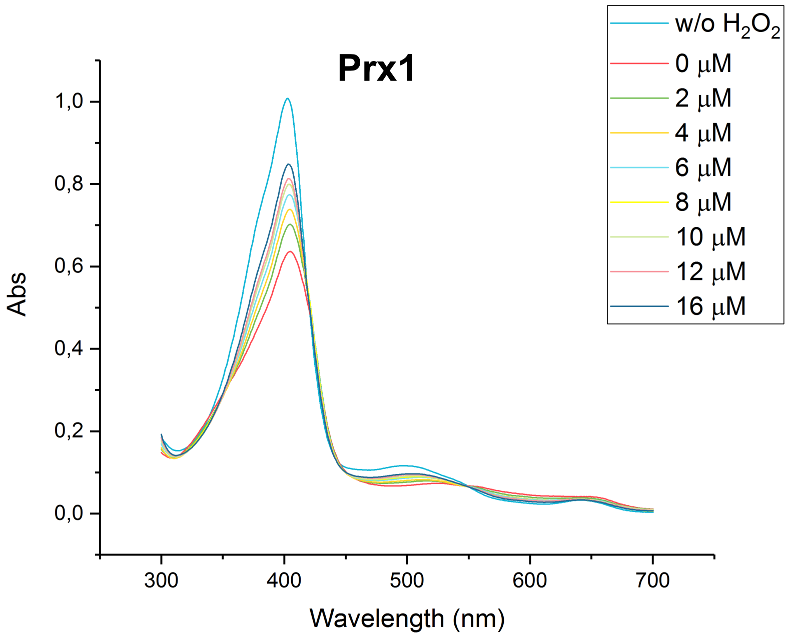

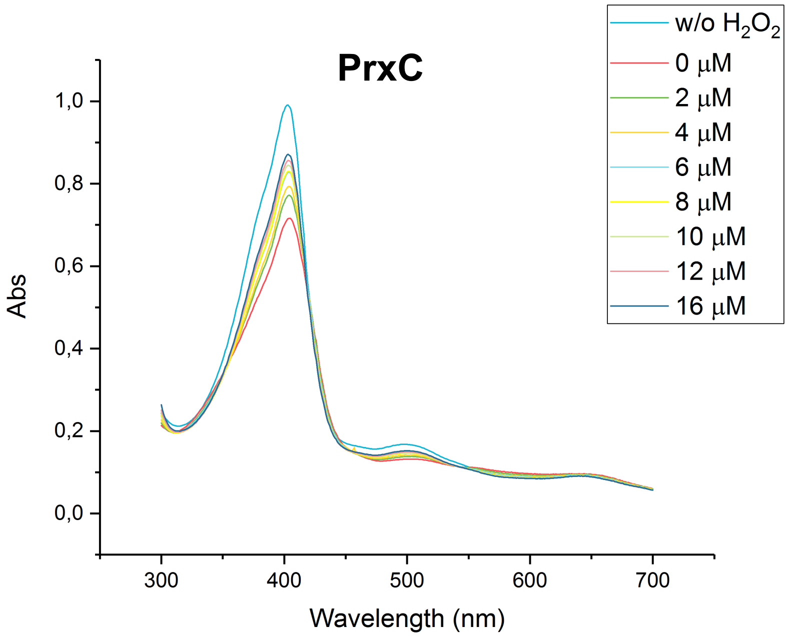

Figure S2. HRP spectra for second-order rate constant determination of the reaction between H_2O_2 and Prx1 and PrxC. The reduced HRP absorbs at 403 nm (w/o H_2O_2 line). After substoichiometric addition of H_2O_2 to ferric HRP, there is a decrease in absorbance at 403 nm due to the formation of HRP-compound I (w/o Prx line). The competitive kinetics occurs after addition of increasing concentrations of Prx1 or PrxC (2, 4, 6, 8, 10, 12 and 16 µM). The higher the Prx concentration, the smaller fraction of HRP becomes oxidized and therefore smaller is the absorption difference comparing to reduced HRP.

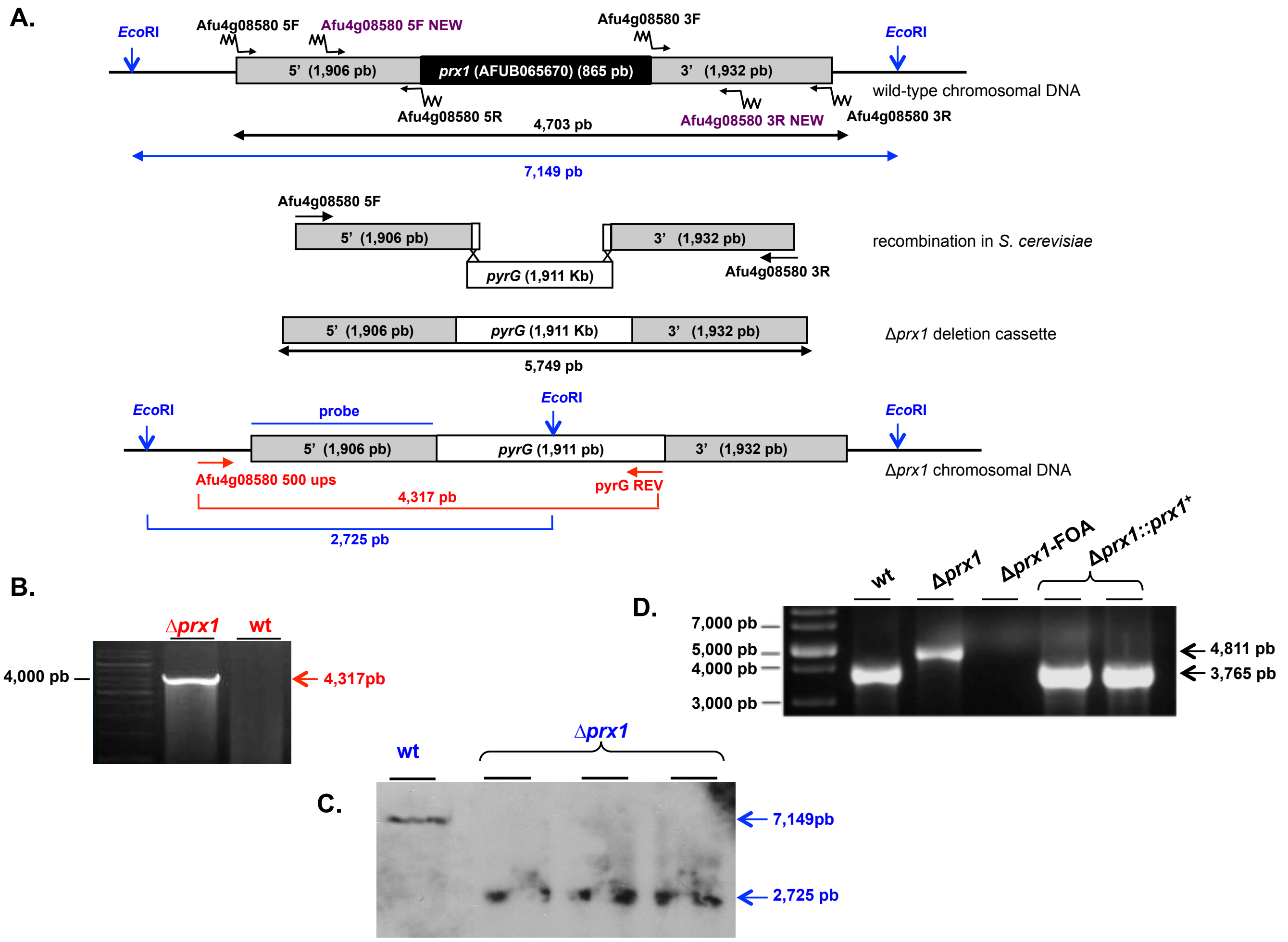

Supplementary Figure S3. Construction of Prx deletion mutants, complementing strains and Prx::GFP tagged strains. Gene replacement strategy for Δ*prx1*, Δ*prxB* and Δ*prxC* deletions. For all mutant strains the *pyrG* gene was used as a selection marker. The primer names and annealing regions are indicated by arrows (primer sequences are described in Supplementary Table S3). The deletion cassettes were constructed by *in vivo* recombination in *S. cerevisiae* (A, E and I). Diagnostic PCR was performed to evaluate the Prx loci after gene replacement using primers located 500 bp upstream of the deletion cassette, as shown by red lines and red letters (B, F and J). Southern blot analysis for each deletion mutant is shown. Each fragment recognized by the probe is indicated by the blue letters and lines (C, G and K). PCR analysis showing the confirmation of each complementing strain (D, H and L). Construction and validation of the double mutant Δ*prxB* Δ*prxC* (M). The Δ*prxC pyrG*⁻ strain was used to transform the Δ*prxB::pyrG* deletion cassette. The *prxB* gene replacement in this mutant was verified by PCR with the primer set Afu5g15070 500 ups and Afu5g15070 3R_NEW (E). Gene replacement strategy for *prx1::gfp*, *prxB::gfp* and *prxC::gfp* strains construction. Each Prx gene without the stop codon was cloned in-frame with the green fluorescent protein (GFP) gene in a C-terminal fusion separated by a Gly-Thr-Arg-Gly spacer (N, P and R). Each corresponding replacement cassette was PCR-amplified and transformed into the *A. fumigatus* wild-type strain. Transformants that integrated the cassette by homologous recombination were tested by PCR with the primer set indicated by the green lines and letters (O, Q and S).

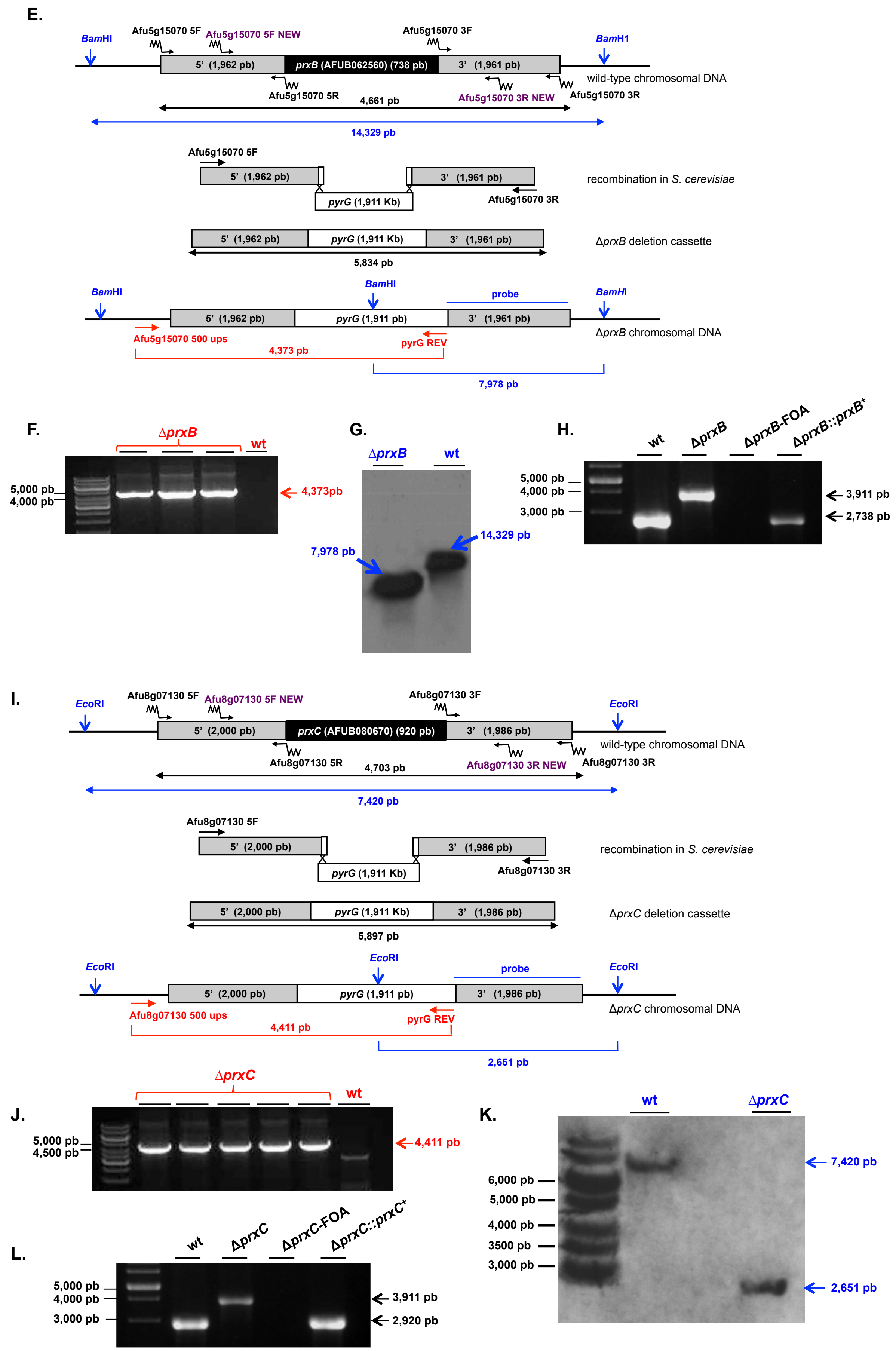

Figure S3: Continuation

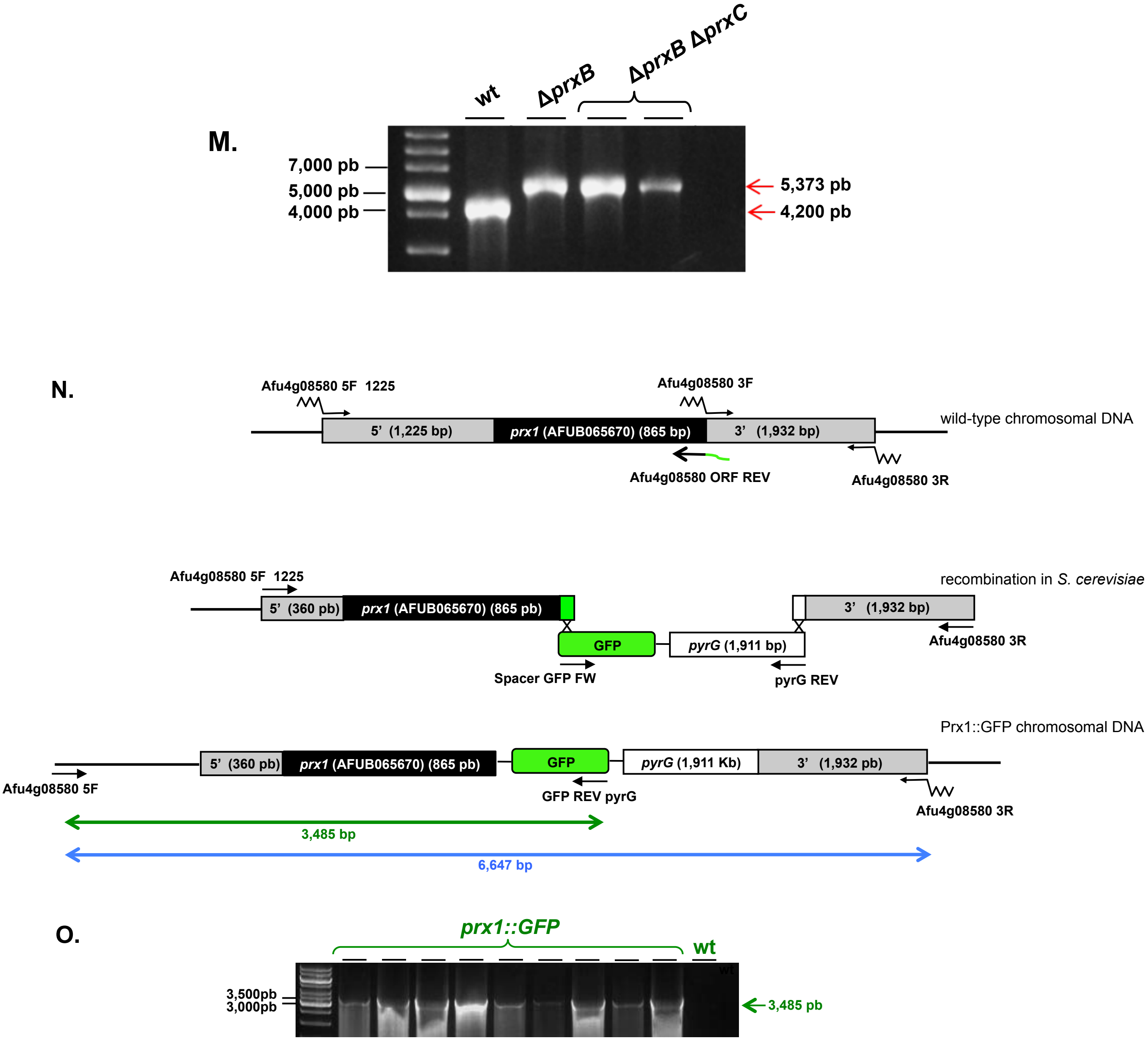

Supplementary Figure S3. Continuation

P.

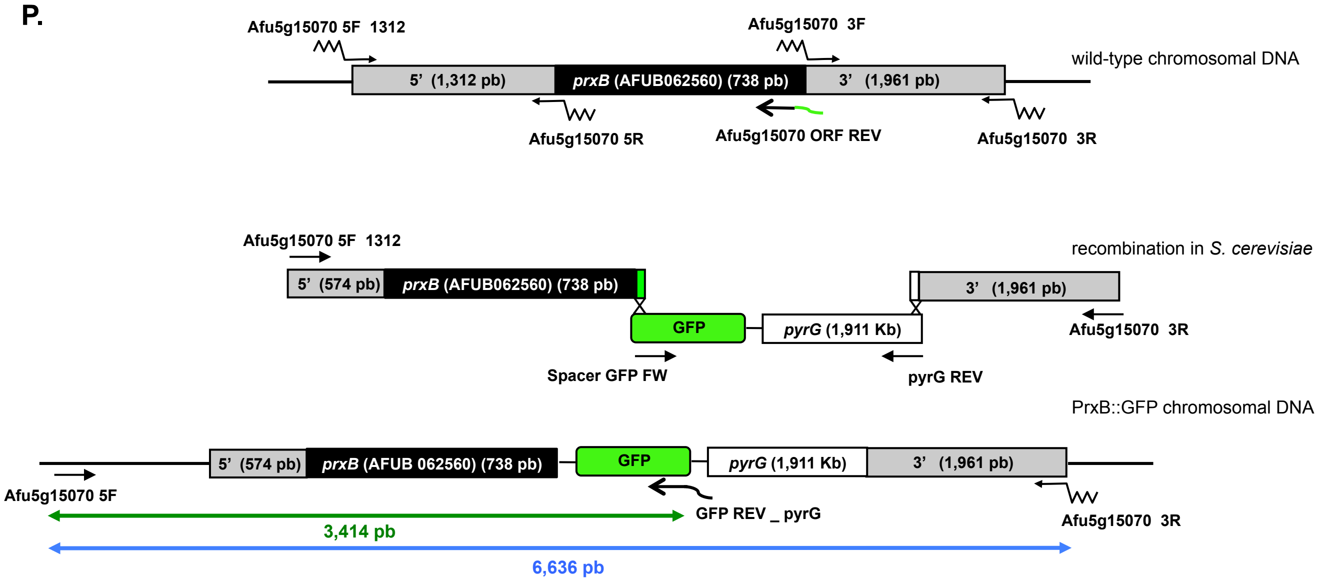

Q.

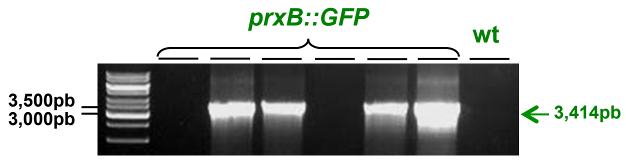

R.

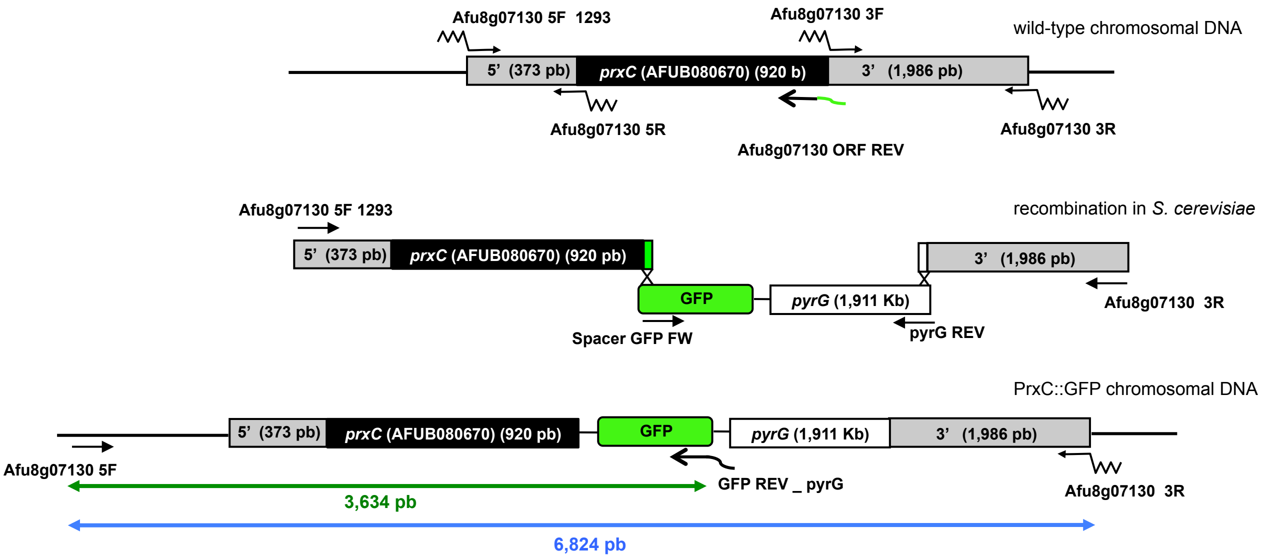

S.

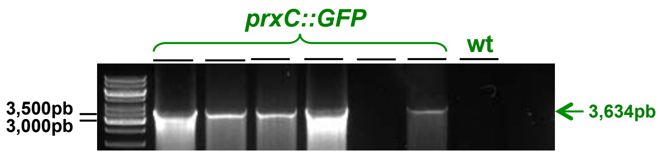

Supplementary Figure S3. Continuation

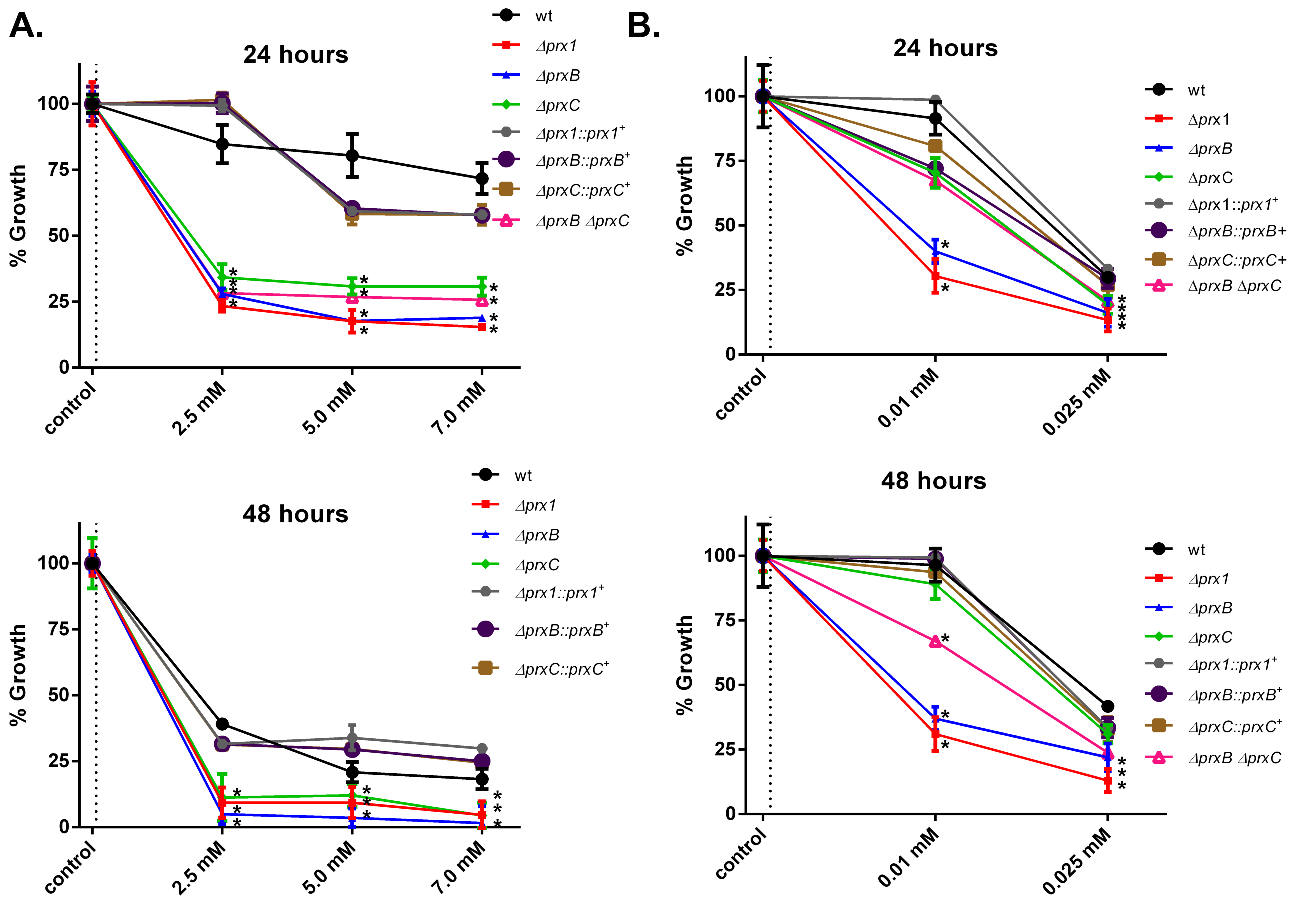

Supplementary Figure S4. *A. fumigatus* 1-Cys Prx deletion mutants are sensitive to oxidants. 1 × 10^4 conidia were inoculated in 200 µl of MM (96 well black plates) supplemented or not with varying concentration of paraquat and menadione and the indicator Alamar blue. Fluorescence intensities (FI) of treated and untreated samples for each strain were obtained after 24 and 48 hours of incubation (37°C). FI were used to calculate the percentage of growth. The results shown are mean ± SD, n = 3. Statistical analysis was performed using a one-way ANOVA with Dunnett's *post hoc* test compared to the control condition (* $p \leq 0.01$). (A) paraquat. (B) menadione.

A.

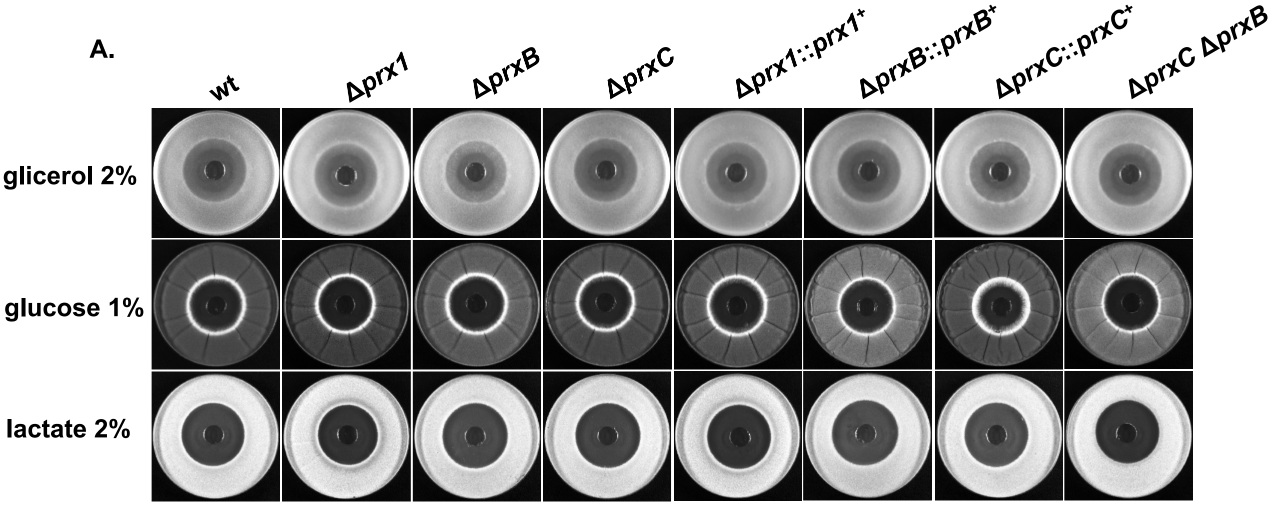

B.

Inhibition diameter (cm) - H_2O_2 8%

**Strain**	**Carbon source in minimal medium**		
	glicerol 2%	glucose 1%	lactate 2%
**wt**	1.6 ± 0.00	1.25 ± 0.00	1.5 ± 0.07
Δ***prx1***	1.7 ± 0.03	1.3 ± 0.00	1.5 ± 0.03
Δ***prxB***	1.6 ± 0.07	1.3 ± 0.00	1.6 ± 0.07
Δ***prxC***	1.6 ± 0.03	1.3 ± 0.03	1.6 ± 0.00
Δ***prx1::prx1***$^{+}$	1.6 ± 0.03	1.35 ± 0.03	1.6 ± 0.07
Δ***prxB::prxB***$^{+}$	1.6 ± 0.03	1.3 ± 0.03	1.55 ± 0.03
Δ***prxC::prxC***$^{+}$	1.6 ± 0.00	1.4 ± 0.07	1.55 ± 0.00
Δ***prxB*** Δ***prxC***	1.55 ± 0.03	1.2 ± 0.03	1.4 ± 0.07

Supplementary Figure S5. The Prx null mutants present wild-type levels of sensitivity to H_2O_2**.** (A) Inhibition zone assay. 1 × 10^7 conidia from each strain were mixed with 1% (w/v) MM top agar and poured onto the MM bottom agar 2% (w/v). In the middle of the top agar plate a well was made and filled with 150 µl of H_2O_2 (2.4 M). Plates were incubated for 24 h at 37°C and the inhibition zones were measured. For this experiment both bottom and top agar layers were supplemented with different carbon sources at the indicated concentrations. (B) Inhibition zone diameters of the wild-type and mutant strains. Values indicate mean ± SD, n = 4 (non-significant, one-way ANOVA).

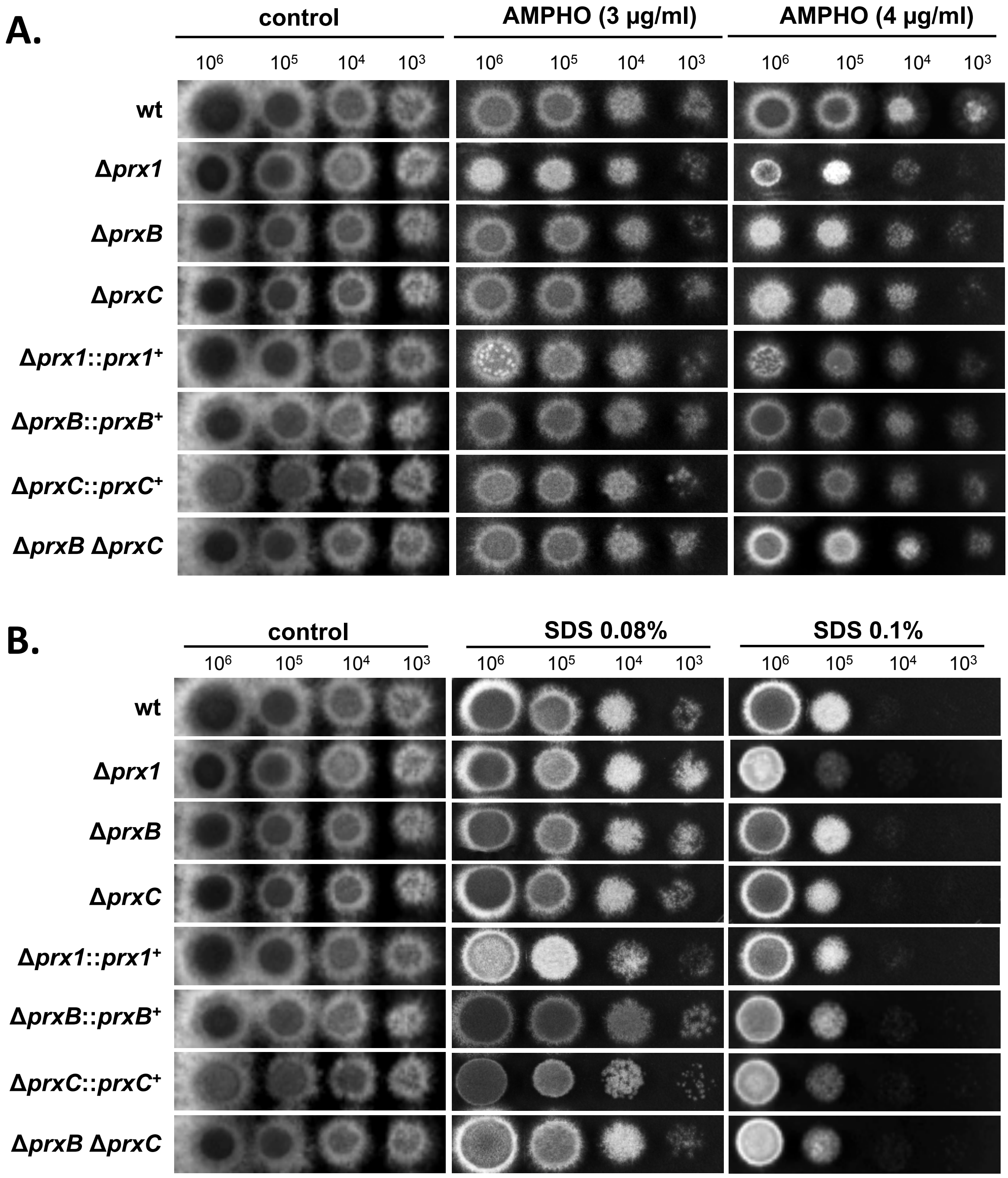

Supplementary Figure S6. Growth phenotypes of Prx mutants in response to antifungal drugs. The indicated numbers of conidia were inoculated onto MM plates that were supplemented with amphotericin B (AMPHO) or SDS. The plates were incubated for 48 h at 37°C and photographed. The results are representative of four independent experiments.

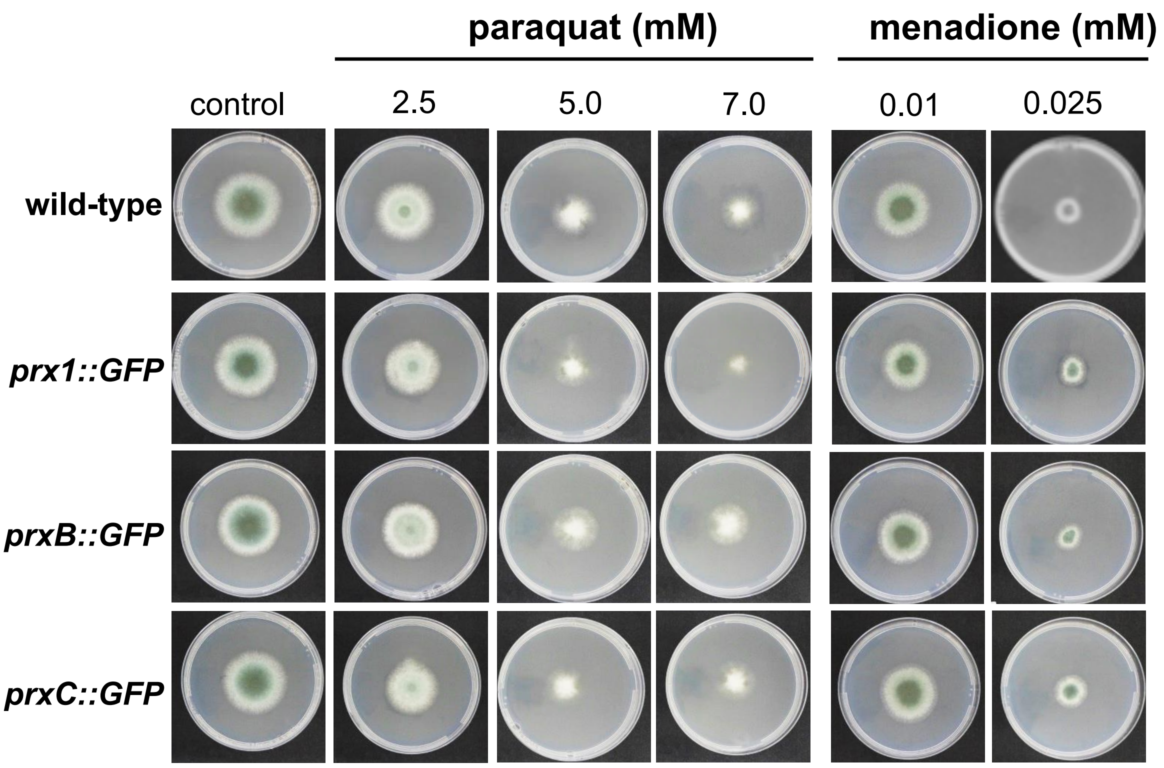

Supplementary Figure S7. Radial growth of the wild-type and Prx::GFP strains in MM. 1 × 10^4 conidia of each strain were inoculated onto the center of solid MM, supplemented or not with different concentrations of paraquat and menadione, and incubated at 37°C for 72 h. Results are representative of triplicate experiments with similar results.

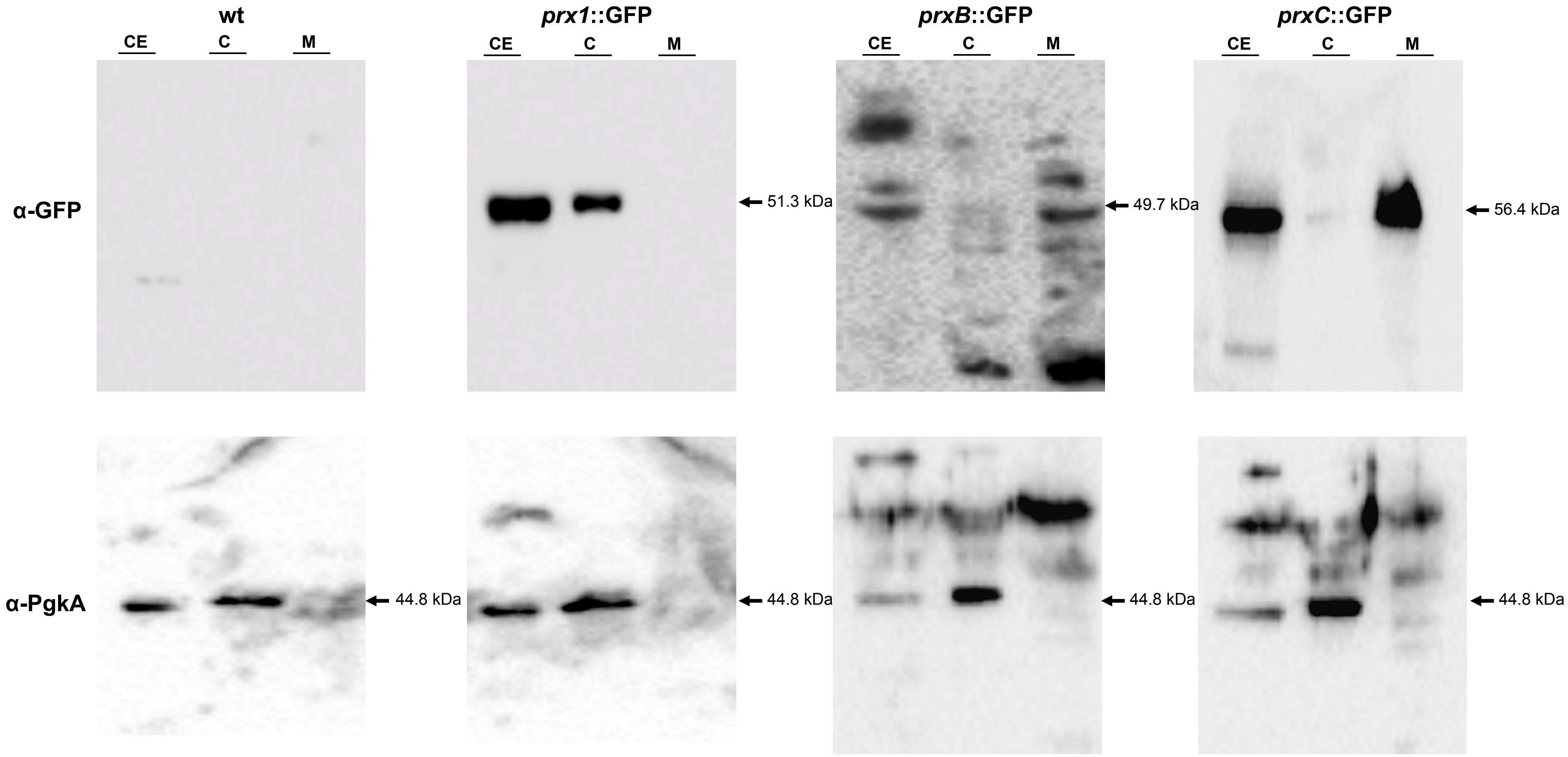

Supplementary Figure S8. Full length blots of Figure 5B.

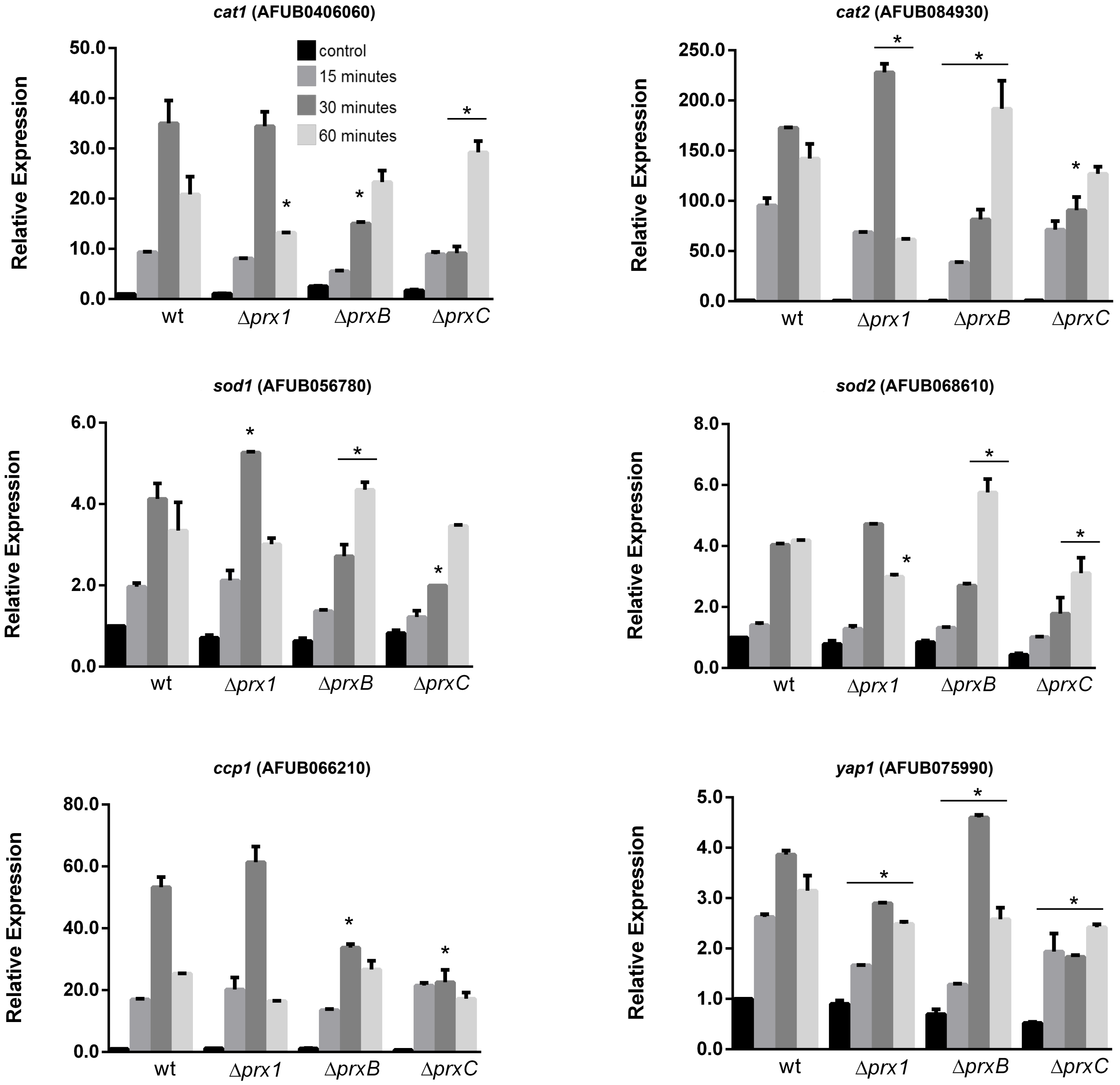

Supplementary Figure S9. Expression of antioxidant genes in wild-type and Prx deletion mutants. The strains were grown in liquid MM for 24 h and treated for 15, 30 and 60 minutes with paraquat (10 mM). The mRNA abundance for each gene was assessed by RT-qPCR and normalized to β-tubulin. The data represent mean ± SD. Experiments were obtained from biological triplicate, each of which was repeated in duplicate in the same run. * $p \leq 0.05$ (one-way ANOVA).

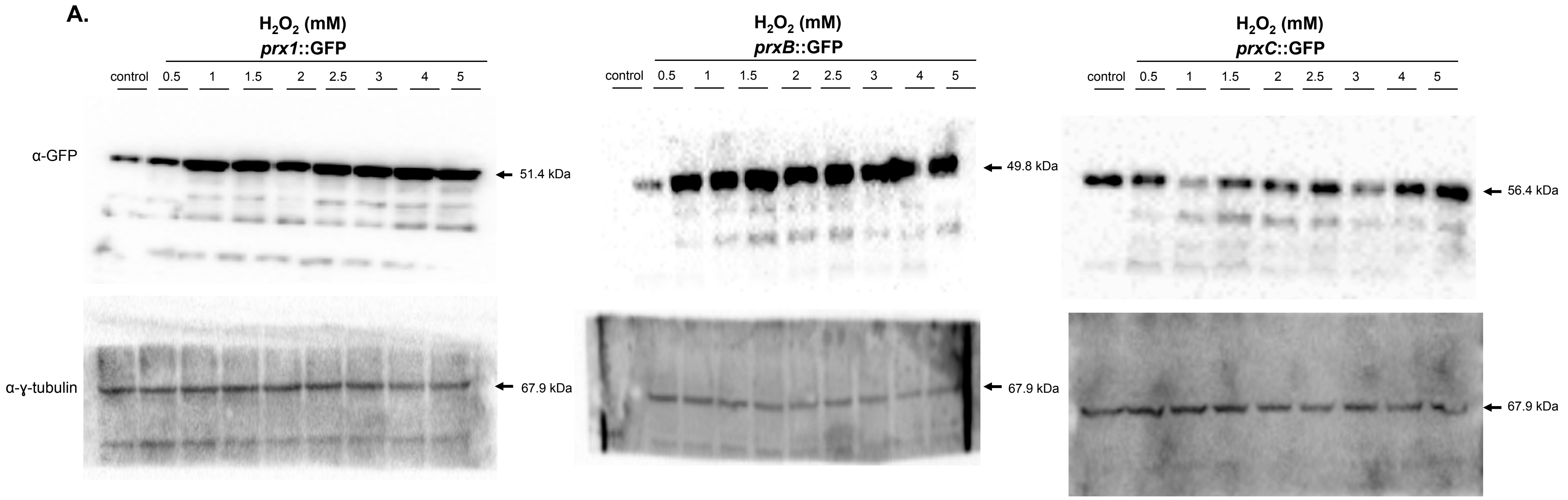

Supplementary Figure S10. Full length blots of Figure 7.

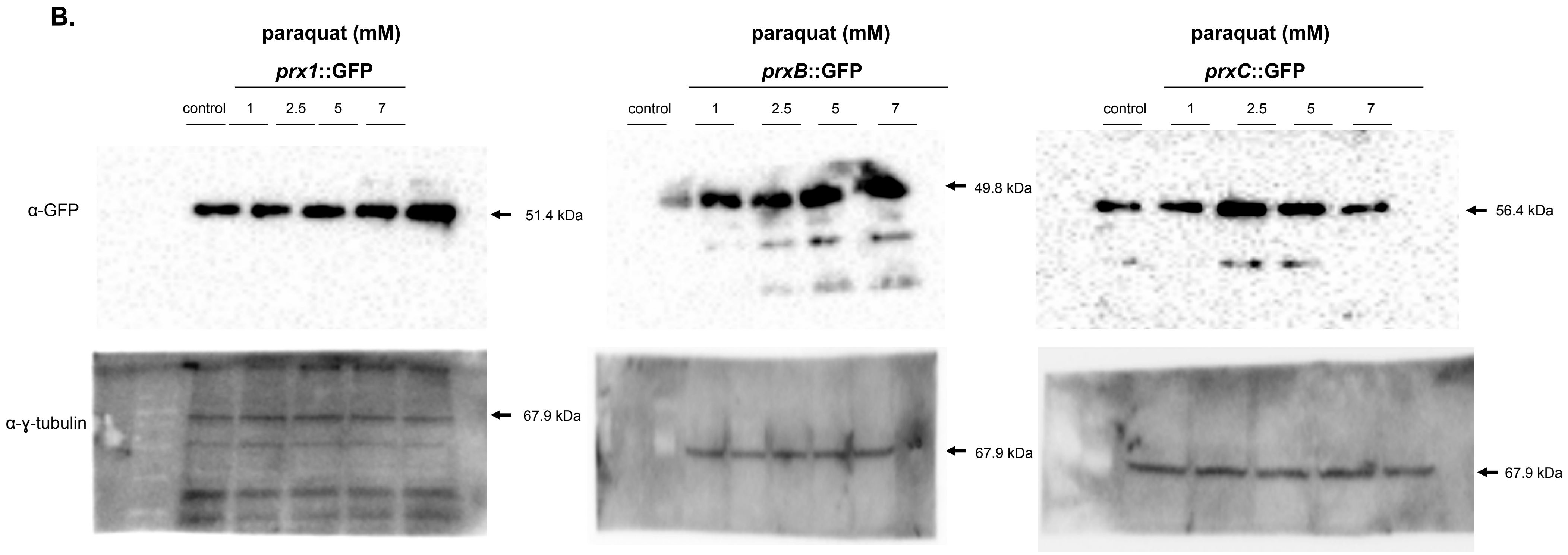

Supplementary Figure S10. Full length blots of Figure 7.

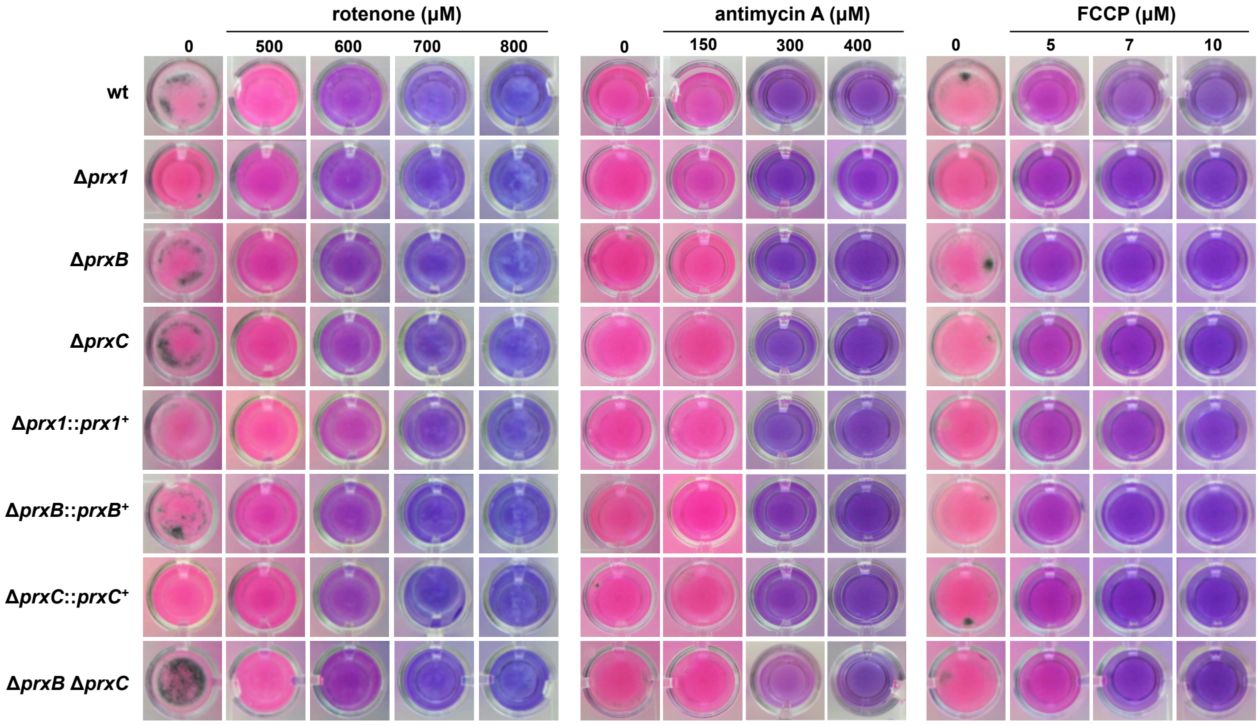

Supplementary Figure S11. Viability of the germlings of the wild-type and Prx mutants in the presence of rotenone, antimicyn A and FCCP. Viability is shown by the indicator Alamar blue. Plates were incubated at 37°C for 72 h and then photographed. A bluish color indicates decreased mitochondrial activity and therefore, lower viability.

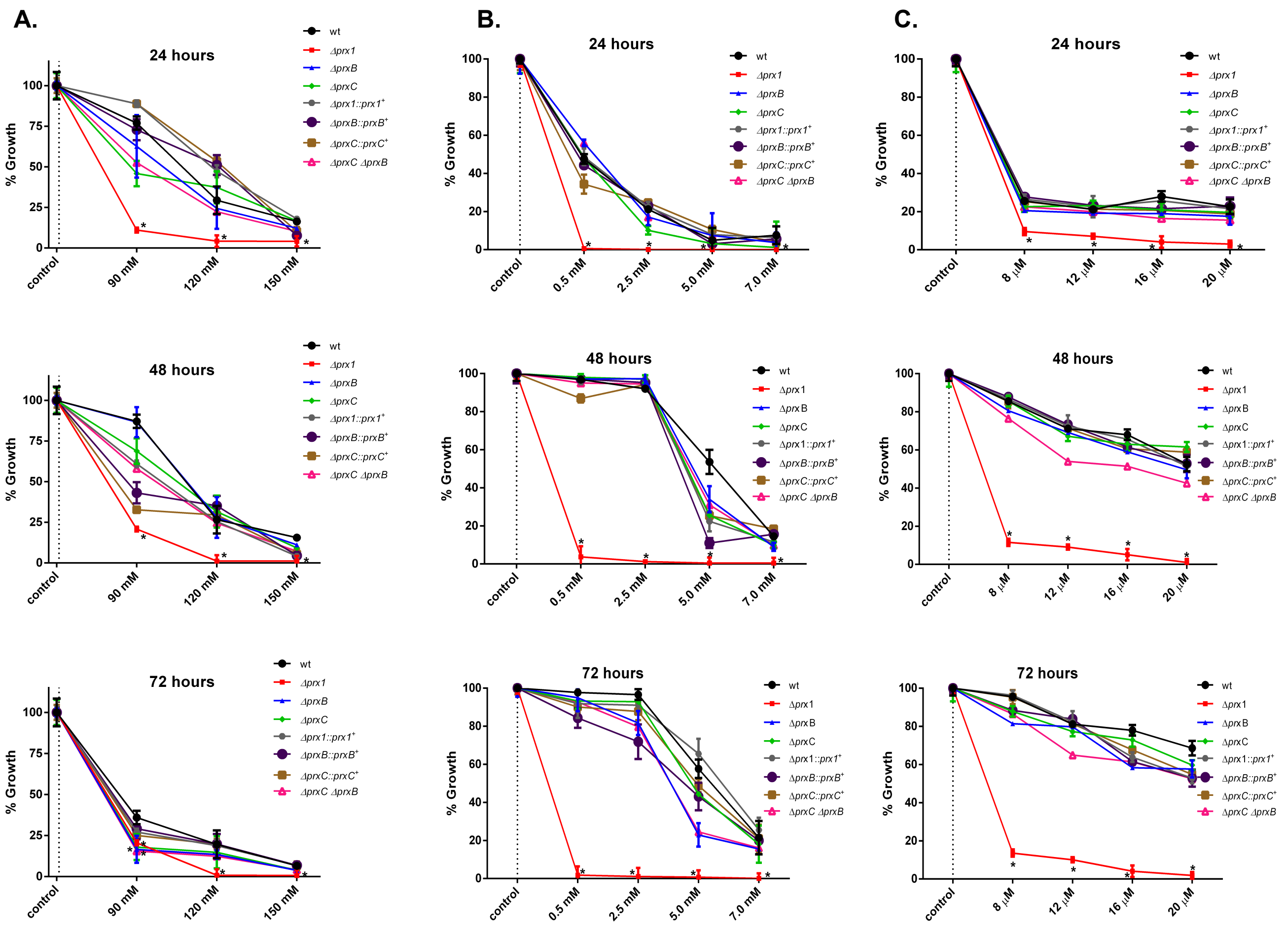

Supplementary Figure S12. Survival curves of 1-Cys Prx in the presence of electron transport chain inhibitors. 1 × 10^4 conidia were inoculated in 200 µl of MM (96 well black plates) supplemented or not with varying concentrations of malonate (A), SHAM (salicylhydroxamic acid) (B) and oligomycin (C) and the indicator Alamar blue. Fluorescence intensities (FI) of treated and untreated samples for each strain were obtained after 24, 48 and 72 hours of incubation (37°C). FI were used to calculate the percentage of growth. The results shown are mean ± SD, n = 3. Statistical analysis was performed using a one-way ANOVA with Dunnett's *post hoc* test compared to the control condition (* $p \leq 0.01$).

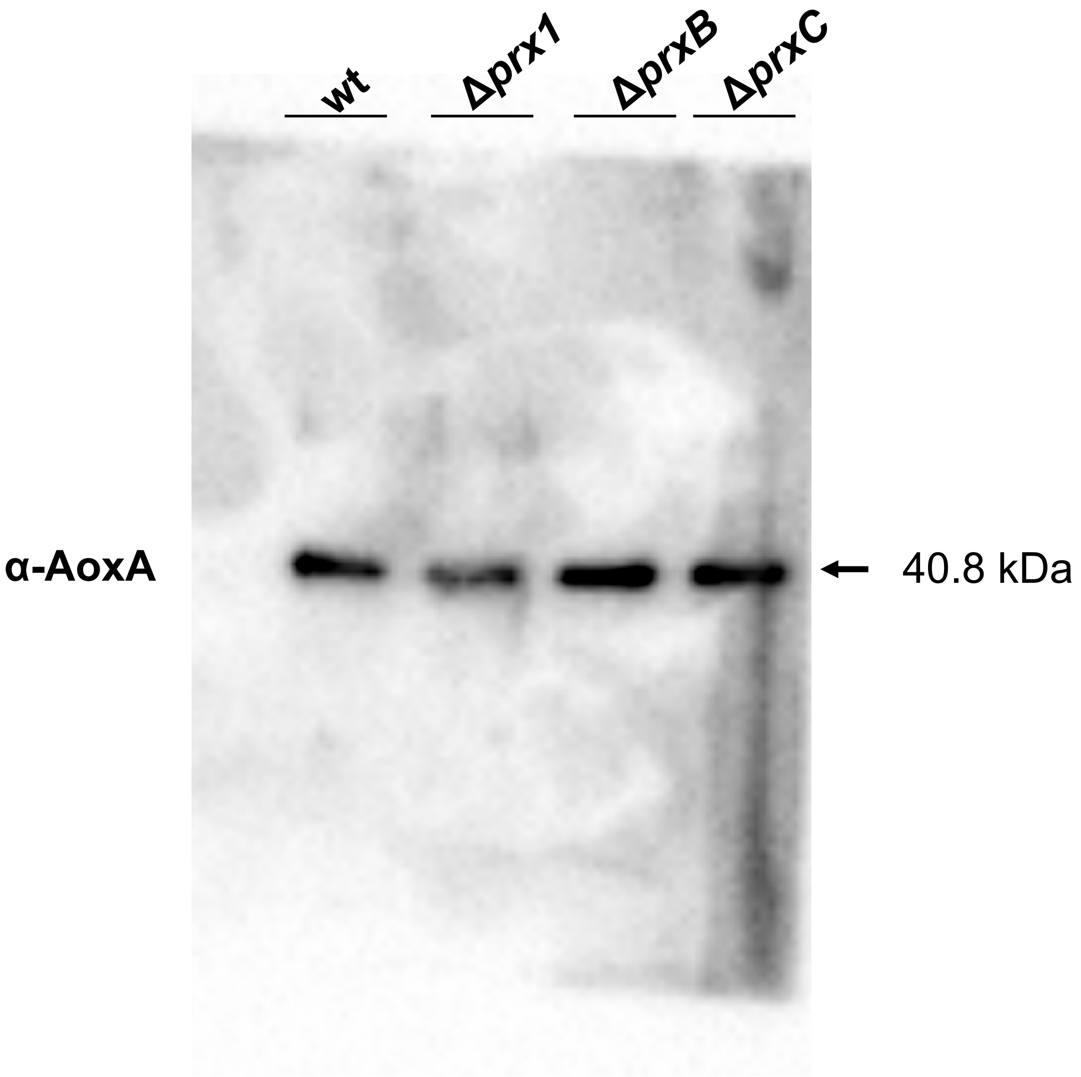

Supplementary Figure S13. Full length blots of Figure 8C.

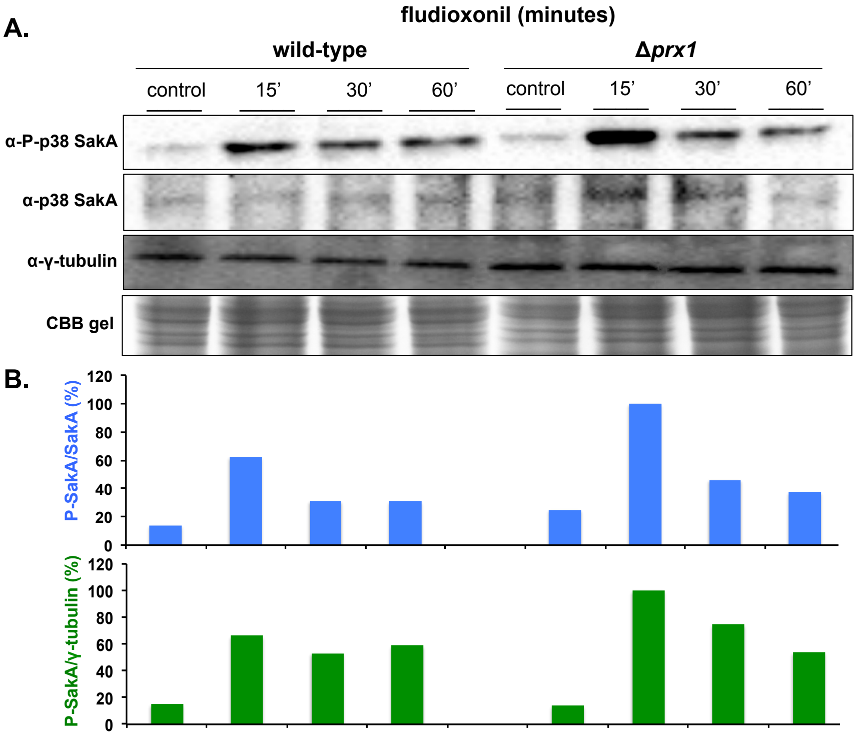

Supplementary Figure S14. Western blotting assay of SakA phosphorylation in response to fludioxonil (1 μg/ml). (A) Anti phospho-p38 and anti-p38 antibodies were used to detect the phosphorylation of SakA and total SakA, respectively. Anti-γ-tubulin antibody was used as a loading control. (B) Signal intensities were quantified using ImageJ software, and the percentage ratios of p-SakA/SakA or p-SakA/ γ-tubulin were calculated in comparison to the untreated control of each strain (blue and green lower graphs, respectively). A Coomassie Brilliant Blue (CBB) stained gel of the protein extract served as an additional loading control.

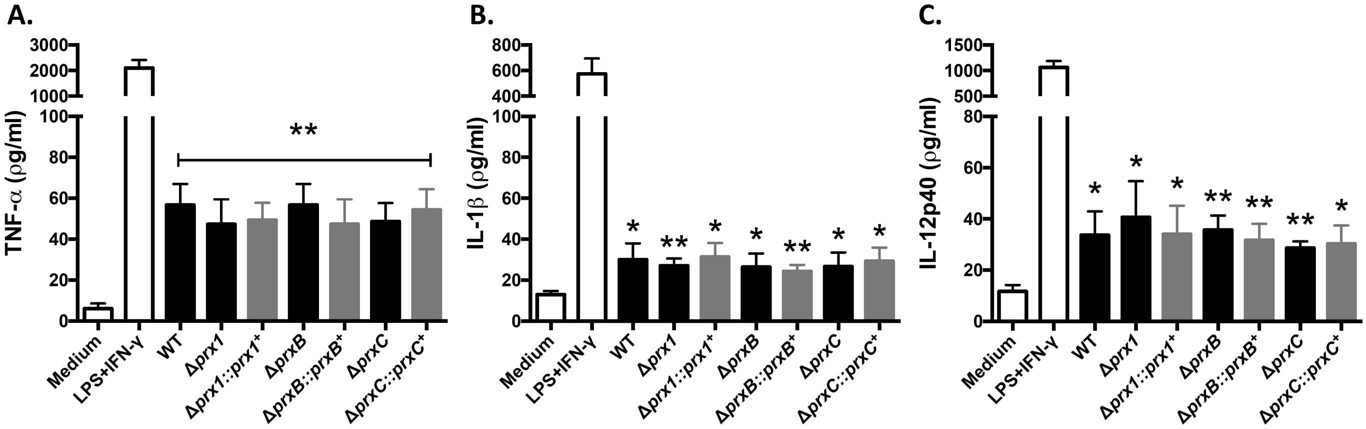

Supplementary Figure S15. Cytokine secretion profile from bone marrow-derived macrophages (BMDMs) co-cultivated with Prx null mutants. BMDMs were obtained from C57BL/6 adult mice. BMDMs were incubated for 24 h with medium, LPS + INF-γ, and *A. fumigatus* strains used in this study. The supernatants were assayed for TNF-α (A), IL-1β (B) and IL-12p40 (C) levels. Data are representative from three separate assays. * $p \leq 0.005$ compared to medium, ** $p \leq 0.01$ compared to medium.

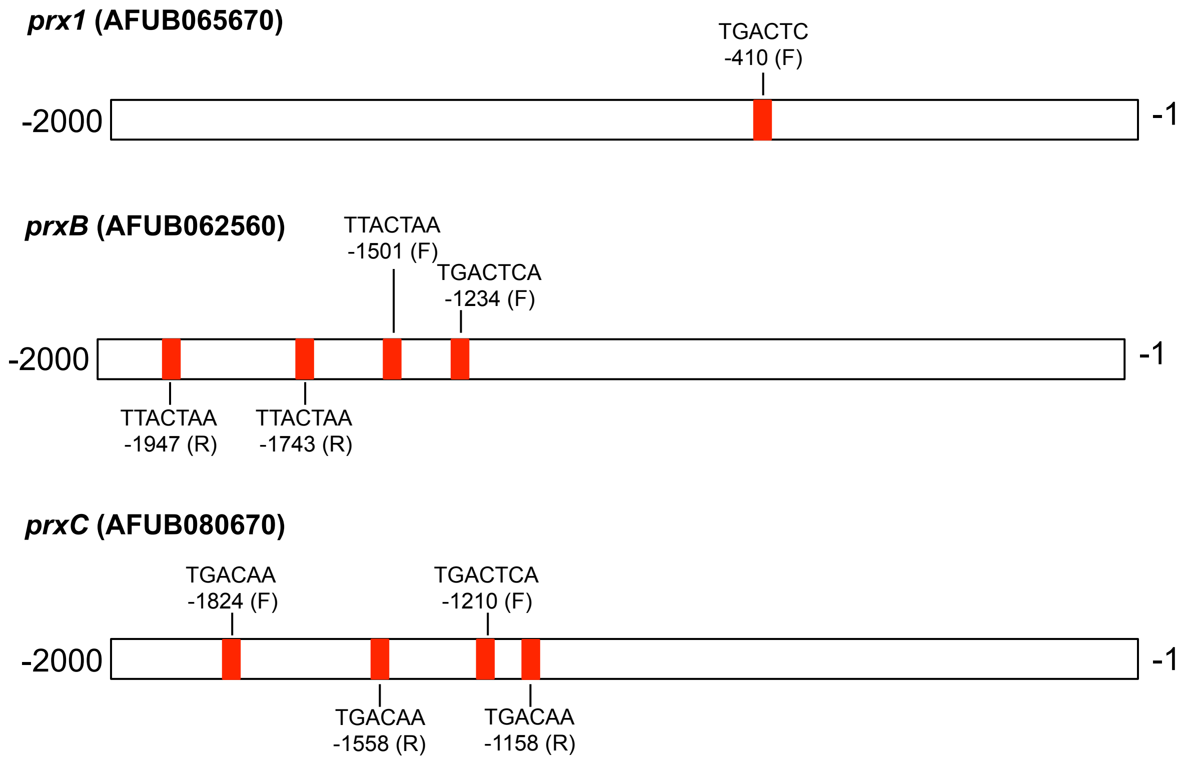

Figure S16: The promoter region of the three 1-Cys Prx presents putative binding sites for the predict Yap1 consensus. The promoter regions (-2000 bp upstream to the initial ATG codon) of *prx1*, *prxB* and *prxC*-encoding genes were screened for potential transcription factors binding sites using the tool available in Yeastract (http://www.yeastract.com)[16,17].

References

1. Sievers, F. *et al.* Fast, scalable generation of high-quality protein multiple sequence alignments using Clustal Omega. *Mol Syst Biol* **7**, 539, doi:10.1038/msb.2011.75 (2011).
2. Waterhouse, A. M., Procter, J. B., Martin, D. M., Clamp, M. & Barton, G. J. Jalview Version 2--a multiple sequence alignment editor and analysis workbench. *Bioinformatics* **25**, 1189-1191, doi:10.1093/bioinformatics/btp033 (2009).
3. Claros, M. G. & Vincens, P. Computational method to predict mitochondrially imported proteins and their targeting sequences. *European journal of biochemistry / FEBS* **241**, 779-786 (1996).
4. Chaveroche, M. K., Ghigo, J. M. & d'Enfert, C. A rapid method for efficient gene replacement in the filamentous fungus Aspergillus nidulans. *Nucleic acids research* **28**, E97 (2000).
5. Malavazi, I. & Goldman, G. H. Gene disruption in Aspergillus fumigatus using a PCR-based strategy and in vivo recombination in yeast. *Methods Mol Biol* **845**, 99-118, doi:10.1007/978-1-61779-539-8_7 (2012).
6. Feng, X. *et al.* HacA-independent functions of the ER stress sensor IreA synergize with the canonical UPR to influence virulence traits in Aspergillus fumigatus. *PLoS pathogens* **7**, e1002330, doi:10.1371/journal.ppat.1002330 (2011).
7. Teepe, A. G., Loprete, D. M., He, Z., Hoggard, T. A. & Hill, T. W. The protein kinase C orthologue PkcA plays a role in cell wall integrity and polarized growth in Aspergillus nidulans. *Fungal Genet Biol* **44**, 554-562, doi:10.1016/j.fgb.2006.10.001 (2007).
8. Sambrook, J. & Russell, D. W. *Molecular Cloning: A Laboratory Manual* 3rd edn, (CSHL Press, 2001).
9. Hagiwara, D. *et al.* NikA/TcsC histidine kinase is involved in conidiation, hyphal morphology, and responses to osmotic stress and antifungal chemicals in Aspergillus fumigatus. *PloS one* **8**, e80881, doi:10.1371/journal.pone.0080881 (2013).
10. Schneider, C. A., Rasband, W. S. & Eliceiri, K. W. NIH Image to ImageJ: 25 years of image analysis. *Nat Methods* **9**, 671-675 (2012).
11. Fedorova, N. D. *et al.* Genomic islands in the pathogenic filamentous fungus Aspergillus fumigatus. *PLoS Genet* **4**, e1000046, doi:10.1371/journal.pgen.1000046 (2008).
12. Krappmann, S., Sasse, C. & Braus, G. H. Gene targeting in Aspergillus fumigatus by homologous recombination is facilitated in a nonhomologous end-joining-deficient genetic background. *Eukaryotic cell* **5**, 212-215, doi:10.1128/EC.5.1.212-215.2006 (2006).
13. da Silva Ferreira, M. E. *et al.* The akuB(KU80) mutant deficient for nonhomologous end joining is a powerful tool for analyzing pathogenicity in Aspergillus fumigatus. *Eukaryotic cell* **5**, 207-211 (2006).
14. Hagiwara, D., Suzuki, S., Kamei, K., Gonoi, T. & Kawamoto, S. The role of AtfA and HOG MAPK pathway in stress tolerance in conidia of Aspergillus fumigatus. *Fungal Genet Biol* **73**, 138-149, doi:10.1016/j.fgb.2014.10.011 (2014).
15. Lessing, F. *et al.* The Aspergillus fumigatus transcriptional regulator AfYap1 represents the major regulator for defense against reactive oxygen intermediates but is dispensable for pathogenicity in an intranasal mouse infection model. *Eukaryotic cell* **6**, 2290-2302, doi:10.1128/EC.00267-07 (2007).

16. Abdulrehman, D. *et al.* YEASTRACT: providing a programmatic access to curated transcriptional regulatory associations in Saccharomyces cerevisiae through a web services interface. *Nucleic Acids Res* **39**, D136-140, doi:10.1093/nar/gkq964 (2011).
17. Teixeira, M. C. *et al.* The YEASTRACT database: an upgraded information system for the analysis of gene and genomic transcription regulation in Saccharomyces cerevisiae. *Nucleic Acids Res* **42**, D161-166, doi:10.1093/nar/gkt1015 (2014).
